# Cathodic hydrodimerization of nitroolefins

**DOI:** 10.3762/bjoc.11.131

**Published:** 2015-07-14

**Authors:** Michael Weßling, Hans J Schäfer

**Affiliations:** 1 Organisch-Chemisches Institut der Westfälischen Wilhelms-Universität, Correns-Straße 40, 48149 Münster, Germany

**Keywords:** cathodic hydrodimerization, C–C bond formation, 1,4-dinitrocompounds, electrosynthesis, nitroalkene

## Abstract

Nitroalkenes are easily accessible in high variety by condensation of aldehydes with aliphatic nitroalkanes. They belong to the group of activated alkenes that can be hydrodimerized by cathodic reduction. There are many olefins with different electron withdrawing groups used for cathodic hydrodimerization, but not much is known about the behaviour of the nitro group. Synthetic applications of this group could profit from the easy access to nitroolefins in large variety, the C–C bond formation with the introduction of two nitro groups in a 1,4-distance and the conversions of the nitro group by reduction to oximes and amines, the conversion into aldehydes and ketones via the Nef reaction and base catalyzed condensations at the acidic CH bond. Eight 1-aryl-2-nitro-1-propenes have been electrolyzed in an undivided electrolysis cell to afford 2,5-dinitro-3,4-diaryl hexanes in high yield. The 4-methoxy-, 4-trifluoromethyl-, 2-chloro- and 2,6-difluorophenyl group and furthermore the 2-furyl and 2-pyrrolyl group have been applied. The reaction is chemoselective as only the double bond but not the nitro group undergoes reaction, is regioselective as a ß,ß-coupling with regard to the nitro group and forms preferentially two out of six possible diastereomers as major products.

## Introduction

**Scheme 1:**
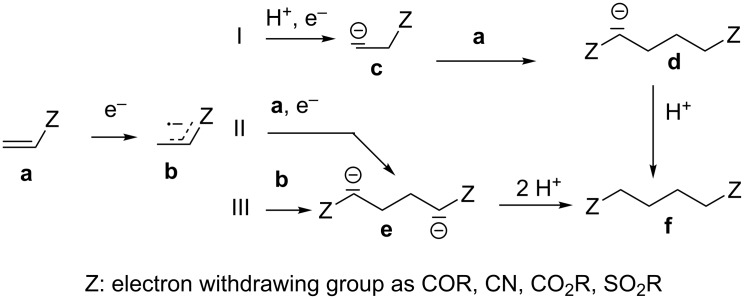
Proposed mechanisms via pathways (I) to (III) for the cathodic hydrodimerization of olefins with electron attracting substituents.

Olefins being activated by an electron withdrawing group can be hydrodimerized by cathodic reduction [1–2]. Thereby, the cathode serves as cheap, versatile, immobilized and mostly non-polluting reagent providing economical and ecological advantages compared to chemical reducing agents [3–4]. Alkenes with a large variety of electron withdrawing groups have been explored in cathodic hydrodimerizations (Scheme 1) [1–2]. We were interested in the nitro group as a substituent. It can be easily introduced by addition of a nitroalkyl anion to a carbonyl group followed by elimination of water from the resulting alcohol. The nitroolefin can be reduced at the nitro group, at the double bond and simultaneously at both groups. In acidic medium the nitro group is reduced between −0.25 V to −0.55 V vs SCE to mixtures of *syn*/*anti*-oximes in 85% to 92% yield at a mercury pool cathode and with slightly lower yields at a graphite cathode [5–8]. The current controlled reduction of alkyl- and aryl-substituted nitroalkenes in acidic medium affords mixtures of ketones and oximes in yields of 39% to 72% [9] and 55% to 91% [10], respectively.

Conducting the reduction at the more negative potential of −1.1 V to −1.3 V vs SCE and otherwise comparable conditions amines are obtained in 60% to 69% yield [5]. Thereby, (*E*)-1-(3-cyclohexen-1-yl)-2-nitroethene can be chemoselectively reduced in 69% yield to 1-amino-2-(3-cyclohexen-1-yl)ethane without hydrogenating the C–C double bond.

The hydrodimerization of nitro olefins should lead to 1,4-dinitroalkanes following the regioselectivity found in other hydrodimerizations of activated olefins [1–2]. Thereby, the proton concentration in the electrolyte should be not too high, as otherwise the reduction of the nitro group to oximes would be favoured. On the other side the electrolyte should not be aprotic as protons are required for protonation of the intermediate anions in the reductive dimerization (Scheme 1).

According to the proposed mechanism for the cathodic hydrodimerization the radical anion **b** formed by one electron reduction of the substrate **a**, has three pathways for dimerization [1–2]. In path (I) protonation followed by one-electron reduction leads to anion **c**, which in a Michael addition with substrate **a** forms the anion **d**, which is protonated to hydrodimer **f**. In path (II) **b** undergoes a nucleophilic addition to **a** forming a dimer radical anion that is reduced to **e** that is then protonated to the dimer **f**. In path (III) the radical coupling of two radical anions **b** leads to the dianion **e**, which is protonated to the product.

We first checked the cathodic reduction of (*E*)-2-nitro-1-phenyl-1-propene (**1**) [for chemical formulas see Table 4] whether the dimer 2,5-dinitro-3,4-diphenylhexane (**2**) can be obtained and which would be the optimal conditions for a high selectivity and yield. The optimization and subsequent hydrodimerization of eight nitro olefins has been previously reported in [6–8]. There have been reports on the reductive dimerization of nitro alkenes prior to 1991. 1,4-Dinitro-2,3-diphenylbutane (**3**) has been obtained in less than 20% yield in the catalytic hydrogenation of β-nitrostyrene (**4**) [11]. Hydrodimerization of **4** was observed in enzymatic reduction [12]. Furthermore **3** was found in the reduction of **4** with TiCl_3_ [13–14]. High dimer yields are reported for the reduction of several nitro olefins with the dianion of cyclooctatetraene [15]. ß-Nitrostyrene (**4**) has been reductively dimerized with organomanganese reagents to **3** in low yield [16]. The electrochemical reduction of 1-nitroalkenes was studied by cyclic voltammetry and controlled potential coulometry. The reduction probably proceeds by initial formation of the radical anion, which subsequently dimerizes [17]. Later conditions were described to achieve selectively either a cathodic ß,ß-coupling (cathodic hydrodimerization) or a α,ß-coupling with aliphatic nitro alkenes having acidic α-protons. ß,ß-Coupling can be achieved in good to high yield (41–95%) at high current densities [18]. In the reduction of 3,3-dimethyl-1-nitrobut-1-ene the intermediate radical anion has been identified by ESR. Nitroalkene **4** is reported to be converted quantitatively to the hydrodimer **3** with SmI_2_ [19]. A catalytic reductive β,β-carbon coupling of nitroalkenes catalyzed by a *N*-heterocyclic carbene has been reported recently. Diastereomers are formed, whose dr (*d,l-* over *meso-*ratio) ranges between 66:34 to 90:10. The interesting new reaction proceeds through a radical anion of the nitroalkene generated in a catalytic redox process. For ß-isopropyl-nitroethylene the radical anion has been identified by ESR [20].

## Results and Discussion

### Investigation of the cathodic hydrodimerization of nitroalkene **1** to hydrodimer **2**

**Scheme 2:**
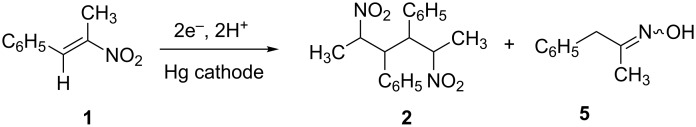
Cathodic reduction of nitroalkene **1** to hydrodimer **2** and oxime **5**.

The cathodic hydrodimerization is performed in a divided electrolysis cell by variation of the electrolyte (Table 1, Scheme 2). The working potential was chosen from cyclic voltammetry and current/voltage curves in the cell used for the preparative conversion. The potential in the controlled potential electrolysis was −0.9 V to −0.95 V vs SCE.

**Table 1:** Hydrodimerization of **1** in dependence on the electrolyte composition.

Nr.	**1** (mmol)	electrolyte	HOAc^a^ (mmol)	*T* (°C)	*Q* (F/mol)	yield (%)^b^		
						**2**	**5**	**1**

1	3.06	DMF/H_2_O (9:1) 0.2 M TBABF_4_	0.2 M	20	1.95	24	20	–
2	3.06	DMF/H_2_O (25:1) 0.2 M TBABF_4_	–	20	1.25	^c^	–	–
3	5.09	DMF 0.2 M TBABF_4_	1 × 5.0	30	1.08	30	7	20
4	7.50	DMF 0.2 M TEA-*p*Tos	2 × 3.7	30	1.5	48	4	–
5	6.13	DMF 0.2 M TEA-*p*Tos	10 × 0.6	30	1.01	60	–	–

^a^0.2 M HOAc in electrolyte (Nr. 1); addition of corresponding fractions of an equivalent of the H^+^-donor at the start (Nr. 3, 4, 5) and after throughput of the respective theoretical charge (Nr. 4, 5). ^b^Isolated by flash chromatography. ^c^Product mixture, about 30% of **2**.

The results indicate: an increased acidity favours the formation of oxime **5** (Table 1, Nr. 1), whilst without a proton donor the olefin presumably is polymerized to a large extent (Table 1, Nr. 2). The addition of acetic acid in portions appears to be a good choice as a too high proton concentration is avoided and the necessary amount of protons is continuously provided in the proper amount. TEA-*p*Tos appears to be a better supporting electrolyte than TBABF_4_: In the latter hydrogen bonds between the fluorine atoms and water possibly increase the water concentration in the double layer and this way reverse partially the hydrophobic effect of the alkyl groups in the tetraethylammonium cation. The dimer yield should increase with increasing radical concentration, which means that at the beginning of the reaction the dimer yield should be higher than towards the end. As olefin **1** and dimer **2** are expected to have a higher oxidation potential than DMF due to the nitro group the advantageous use of an undivided cell appears to be possible. Taking the optimal conditions of electrolysis Nr. 5 in Table 1 the influence of the parameters mentioned above was investigated (Table 2).

**Table 2:** Hydrodimerization of **1**^a^ in dependence of temperature, conversion and cell type.

Nr.	*T* (°C)	*Q* (F/mol)	Yield **2** (%)^b^

5^c^	30	1.01	60
6	50	1.19	63
7	−10	1.27	70
8	0	1.19	81
9	0	0.51	46 (83)^d^
10^e^	30	1.42	44
11^f^	0	0.98	88

^a^5.03 mmol **1** in 25 mL 0.2 M TEA-*p*Tos/DMF. ^b^Isolated yield. ^c^Nr. 5 in Table 1 is shown for comparison. ^d^Yield in parenthesis based on conversion; 45% reisolated **1**. ^e^Undivided cell, 0.25 equiv HOAc. ^f^Undivided cell without addition of acetic acid.

The influence of the temperature is less significant than expected. The increase of the temperature to 50 °C shows a marginal increase of the yield, whilst a temperature decrease is more successful. Best results could be achieved at 0 °C. The yield after 50% charge consumption based on conversion is insignificantly higher (Table 2, Nr. 9). This indicates that there is no higher yield at higher substrate concentration in the first half of the reaction compared to the second half. However, a remarkable increase of the yield is obtained in an undivided cell without addition of a proton donor. With a quantitative conversion of **1** the dimer **2** is obtained in 88% material yield and 90% current yield. Presumably the protons are generated at the anode by oxidation of residual water and/or the solvent DMF. A major source of residual water could be the very hygroscopic tosylate as one of the reviewers suggested. The conditions of Nr. 11 in Table 2 should be suitable for the conversion of further nitroalkenes.

The cyclovoltammogram (CV) of **1** shows two irreversible reduction peaks at −1.08 V and −1.8 V vs SCE. The second peak can be attributed to the reduction of hydrodimer **2**, as for isolated **2** the reduction peak is found at this potential. The first peak can be assigned to the reduction of **1** forming the radical anion. Addition of acetic acid shows no potential shift but a slight increase of the peak current. This could indicate that the radical anion is fast protonated and the resulting radical is further reduced. Proton addition, however, could also favour the reduction of the nitro group to the oxime, which consumes four electrons. Decreasing hydrodimer yields with increasing temperature could be due to the existence of chemical side reactions of the radical anion, such as oligomerization or protonation, which are more accelerated at higher temperatures compared to the radical dimerization. It should be mentioned that at the cathode deep red species are formed that become colorless upon addition of acetic acid. In an undivided cell and an unstirred electrolyte, which allows diffusion between the electrodes, a red colour appears at the cathode, which disappears at the anode. This indicates the formation of coloured nitroalkyl anions and their decolourization by protonation.

**Scheme 3:**
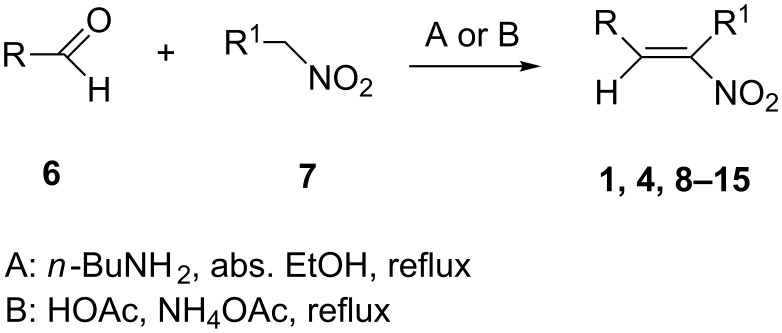
Preparation of the 1-aryl-2-nitroalkenes **1**, **4**, **8–15**.

The nitroalkenes were obtained by condensation of aldehydes with nitroalkanes (Scheme 3, Table 3) [21–22].

**Table 3:** Preparation of 1-aryl-2-nitroalkenes.

Aldehyde	Nitroalkane	Method	Nitroalkene^a^	Yield (%)^b^

**6a**, R: phenyl	**7a**, R^1^: H	^c^	**4**	50^c^
**6a**, R: phenyl	**7b**, R^1^: Me	A	**1**	54
**6a**, R: phenyl	**7c**, R^1^: Et	A	**8**	62
**6b**, R: 4-methoxyphenyl	**7b**, R^1^: Me	B	**9**	44
**6c**, R: 4-trifluoromethylphenyl	**7b**, R^1^: Me	^d^	**10**	42^d^
**6d**, R: 2-furyl	**7b**, R^1^: Me	A	**11**	75
**6e**, R: 2-pyrrolyl	**7b**, R^1^: Me	A	**12**	10^e^
**6f**, R: 2-chlorophenyl	**7b**, R^1^: Me	A	**13**	58
**6g**: 2,6-dichlorophenyl	**7b**, R^1^: Me	B	**14**	36
**6h**: 2,6-difluorophenyl	**7b**, R^1^: Me	A	**15**	62

^a^For the structures of the nitroalkenes see Scheme 4. ^b^Isolated, not optimized yield. ^c^Ref. [23]. ^d^Ref. [24]. ^e^Crude yield higher, product decomposes slowly during recrystallization.

For work-up unreacted aldehyde was removed by way of the bisulfite adduct, this facilitated the crystallization and improved the yields. The preparation of the nitroalkenes **1**, **4**, **8**, **9** is described in [25]; the IR, ^1^H NMR, and MS data are provided in the experimental part (Supporting Information File 1). From a comparison of the experimental δ value for the vinylic proton with this from an increment calculation the cis position of the hydrogen atom to the nitro group can be assigned for the nitroalkenes **1**, **8**, **9**, which is the *E*-configuration.

As the trifluoromethyl compound **10** is not accessible by the method A or B it is prepared in two steps from aldehyde **6c** via the *n*-butylazomethine [24]. Particularly difficult was the synthesis of **12**, where a product mixture is formed; additionally **12** decomposes partly during purification by fractional crystallization, furthermore it is air sensitive. All that leads to low yields of **12**. The dinitrodiene **16** was prepared from 1,4-dinitrobutane and two equivalents of benzaldehyde with 1,2-diaminoethane as catalyst in 59% yield [26]; 1,4-dinitrobutane was prepared from 1,4-dibromobutane [27]. The structures of the prepared compounds were secured by comparing the melting points with these from the literature [24–27] and their spectroscopic data. The nitroolefins **10**–**15** exhibit the same spectroscopic features as these of **1**, **4**, **8**, **9**. The C,H,N and C,H,F,N analyses additionally confirm the structures. From the ^1^H NMR spectra for all nitro olefins the *E*-configuration of the double bond can be derived.

### Cyclic voltammetry

**Scheme 4:**
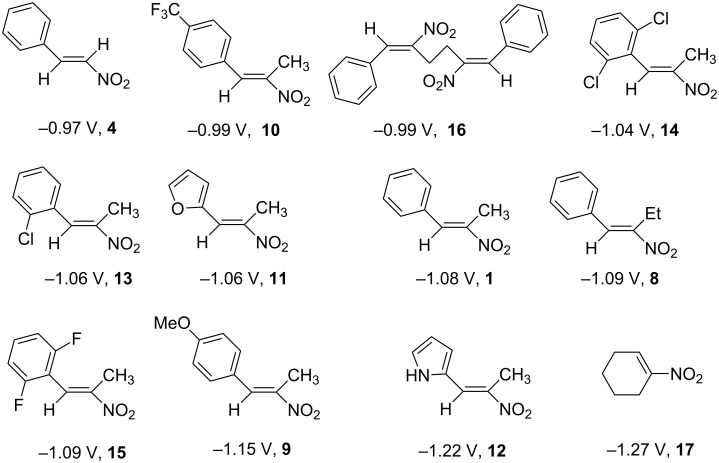
Reduction potentials (*E*_p,c_ in Volt) of nitroolefins. Conditions: amalgamated gold wire, *v* = 0.1 V/s, 0.2 M TEA-*p*Tos in DMF, accuracy of *E*_p,c_ = +/− 0.02V vs SCE, measured against the Marple electrode and converted to SCE.

The reduction potentials (*E*_p,c_) of the nitroalkenes were determined by cyclic voltammetry. The values, ordered by decreasing potentials, are shown in Scheme 4.

The reduction potentials *E*_p,c_ are determined by the conformation of the aryl group, the electron density at the double bond and the nitro group, the energy of the radical anion and the reactivity of the radical anion. We have not determined these values, but have concentrated ourselves on the preparative aspects of the cathodic hydrodimerization. Certain influences of substituents on the reduction potentials of the nitroolefins can be qualitatively seen. Aryl substituents shift the potentials to more negative values according to their Hammett σ values [28]: **10** (4-CF_3_, σ_p_ = 0.53) > **1** (4-H, σ_p_ = 0) > **9** (4-CH_3_O, σ_p_ = −0.12). With a Hammett equation for an electrochemical reaction and using the *E*_p_ values as *E*^0^-values one obtains from these three values a Hammett reaction constant ρ = 5.34. This is similar to the reaction constant ρ = 6.37 obtained from the Hammett plot for the one-electron reduction of substituted benzo- and naphthoquinones in DMF [29], which have an electrophore being similar to this of the nitroolefins. For the other substituents no σ-values are available to apply the Hammett equation. They are ordered according to decreasing *E*_p_-values in three groups: **11** (2-furyl) > **9** (4-CH_3_O) > **12** (2-pyrrolyl); **14** (two *o*-Cl) ≈ **13** (one *o*-Cl) > **15** (two *o*-F); another correlation concerns the vinyl substituents at C2 of the double bond: **4** (2-H) > **1** (2-CH_3_) ≈ **8** (2-C_2_H_5_) > **17** (no aryl group, methylene groups only). These orders are compatible with the electron donating abilities of the substituents, being derived from their σ_m_ values [28]. The more positive potential of **16** compared to **1** could be due to intramolecular interactions of the nitro groups with the non-conjugated double bonds. The *E*_p,c_ of **1** and **4** measured at an amalgamated gold-wire electrode in DMF are somewhat more positive than those measured at a Pt-disc in ACN [17].

**Figure 1:**
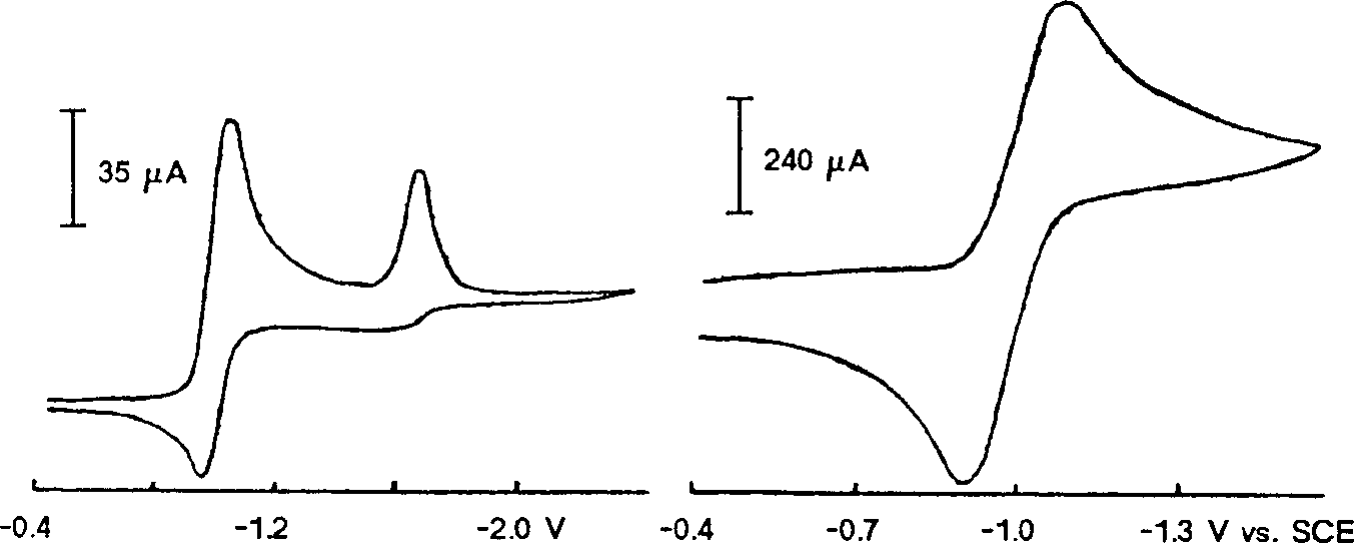
(a) CV of **15**; *v* = 0.1 V/s, (b) CV of **15**; *v* = 10 V/s.

In the CV of all nitroolefins a second reduction peak appears at a potential being 600–800 mV more cathodic compared to the first one. Possibly this is the reduction of the hydrodimer as the CV of the hydrodimer of nitroolefin **1** indicates. This is different for the nitroolefins **14** and **15**, which are *o*,*o*’-disubstituted at the phenyl ring (Figure 1).

In the CV of **14** and **15** (Figure 1a) already at low scan rates (0.1 V/s) an anodic peak (*E*_p,a_ = −0.95 V for **14** and *E*_p,a_ = −0.92 V for **15**) appears in the reverse scan. Reversing the scan after the first peak leads for **15** at a scan rate of 10 V/s to a CV peak with *E_p,c_* = −1.084 V, *E_p,a_* = −0.904 V and *i*_p,c_/*i*_p,a_ = 1. For **14** higher scan rates were necessary to achieve a similar effect, but there the curve became strongly distorted possibly due an increasing capacitive current and iR-drop. This indicates, that most probably due to the *o*,*o*-substituents in **15** the follow-up reaction of the radical anion is slowed down for steric reasons. For **14** no dimer was found (see below). Further electroanalytic investigations were omitted in favour of the preparative scale hydrodimerizations of the nitroolefins.

### Preparative scale electrolyses at the Hg cathode

The preparative scale electrolyses were performed using the following conditions: Hg cathode, undivided cell, 0.2 M TEA-*p*Tos in DMF at 0 °C, cathode potential of −0.90 V to −0.95 V vs SCE. These conditions were optimal for the potential controlled conversion of nitroolefin **1** into dimer **2**. The conversions shown in Table 4 consumed one charge equivalent (Q = 1 F mol^−1^) for completion, then the electrolysis current had decreased to nearly 0 mA. In the work-up following the electrolysis the products in general can be extracted by nonpolar petroleum ether/diethyl ether mixtures from the aqueous emulsions or suspensions, respectively. The insoluble dimer **3** was isolated by filtration and washing the solid with petroleum ether/diethyl ether. The products are obtained after purification by flash chromatography as colourless oils, which are mixtures of diastereomers. They crystallize partially or completely after some time and are in general not sensitive against air and light. An exception is the pyrrole derivative **22**, in the air its light colour deepens quickly to brown. The electrolyses proceed uniformly. The current reaches after a short induction period (1–3 min) depending on the substrate a maximal current of 250–450 mA, which then decreases exponentially to zero.

**Table 4:** Preparative hydrodimerization of nitroalkenes.

Nitroalkene	Hydrodimer^a^	Yield (%)^b^

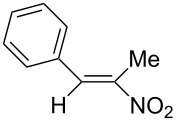 **1**	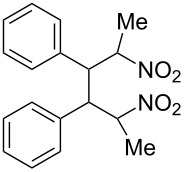 **2**	88
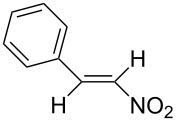 **4**	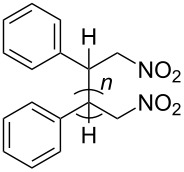 **3**	71^c^
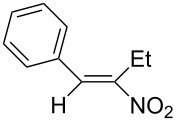 **8**	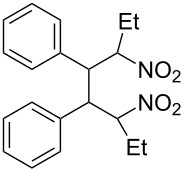 **18**	84
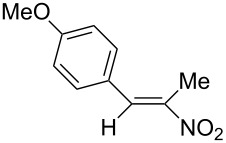 **9**	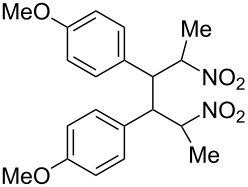 **19**	75
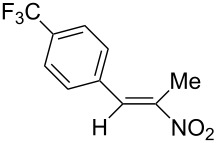 **10**	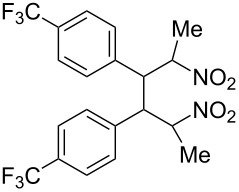 **20**	68
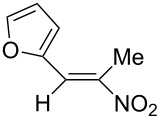 **11**	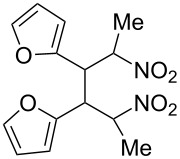 **21**	85
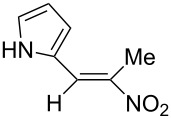 **12**	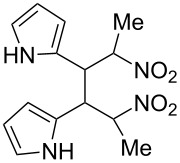 **22**	73^d^
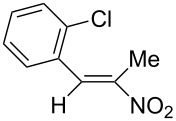 **13**	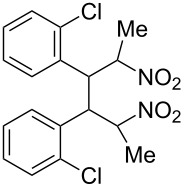 **23**	83

^a^Products are mixtures of diastereomers (see chapter: Structure of the hydrodimers). ^b^Isolated yield; material yield corresponds to 95–100% of the current yield. ^c^See text following Table 4. ^d^Reduction in divided cell, as product is sensitive to anodic oxidation; yield in undivided cell: 60%.

**Scheme 5:**
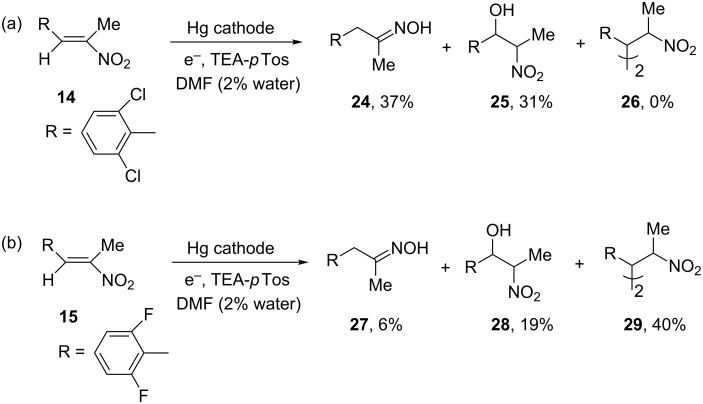
Hydrodimerization of nitroalkene **14** and **15**.

The dichloro derivative **14** deviates from this behaviour. Applying the usual electrolysis conditions no dimer **26** but only the oxime **24** (37%) and the nitro alcohol **25** (31%) are formed (Scheme 5a). As already indicated in the CV of **14** the dimerization of the intermediate radical anion of **14** is apparently hindered for steric reasons, which can explain the absence of the dimer. This can favour the further reaction of the radical anion of **14** to the oxime **24** and the Michael addition of hydroxy ions to form the nitro alcohol **25**.

The smaller space filling of the fluorine atom compared to the chlorine atom should lead to a sterically less hindered radical anion in the reduction of **15**, which allows the formation of 40% of the dimer **29** and leads to less oxime and nitro alcohol as side products (Scheme 5b).

The dimers **2** and **18**–**23** could be identified by ^1^H, ^13^C NMR, MS and elemental analyses (see Structures of the hydrodimers and Experimental part in Supporting Information File 1). Dimer **3** is insoluble in common solvents at rt, thus no ^1^H and ^13^C NMR could be obtained. It has a correct elemental analysis and the IR spectrum is similar to this of **2** and the other dimers with regard to the NO_2_ group. It melts at 238–242 °C with decomposition, which is similar to the product obtained by hydrogenating dimerization of olefin **4** in [11]. The insolubility and the melting point disagree, however, with compound **3** (*n* = 1) described in [20]. From the laser desorption ionization (LDI) mass spectrum of **3** it could be presumed that **3** is mainly a trimer (**3**, *n* = 2). The trimer could arise by a Michael addition of the intermediate dimer radical anion or dimer dianion of **4** to olefin **4**. Indications to greater portions of **3** (*n* = 1) and **3** (*n* = 3) were not found in the LDI–MS. Support for this assumption comes from coulometry for **4** in [17], which indicates oligomerization. Oligomerization does not occur if the substituent α to the nitro group is an alkyl group as in olefin **1**, possibly due to steric hindrance.

**Scheme 6:**
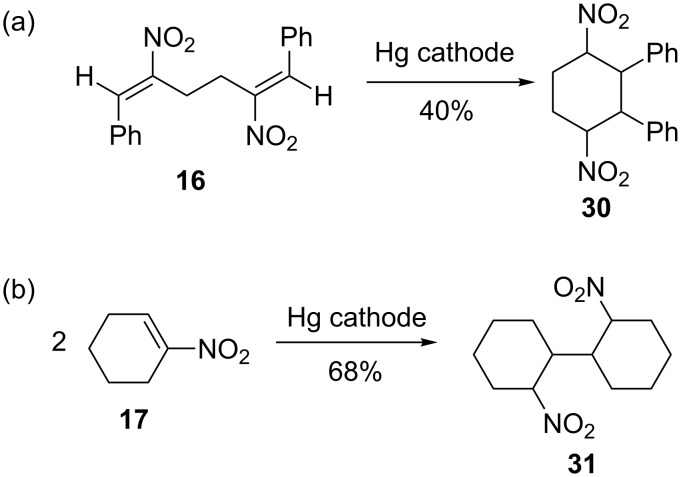
(a) Intramolecular hydrocoupling of dinitrodiene **16** and (b) hydrodimerization of 1-nitrocyclohexene (**17**).

The dinitrodiene **16** is intramolecularly coupled at the Hg cathode to form the dinitrocyclohexane **30** (Scheme 6a), it also does not show the usual behaviour found in the preceding electrolyses. A significant decrease of the current is only found after a current consumption of 2.96 F/mol. Except for benzaldehyde no further side products were detected. **30** is formed as mixture of diastereomers, which could not be separated by flash chromatography.

The aliphatic nitroalkene **17** could be hydrodimerized in 68% yield to the hydrodimer **31**, which is a mixture of diastereomers. Partial separation by flash chromatography and ^1^H NMR spectroscopy of the fractions indicates four diastereomers in a ratio of about 38:9:14:1.

It is possible to substitute the cathode material mercury against the environmentally benign graphite. At a graphite cathode the nitroolefin **1** could be hydrodimerized to the dimer **2**. With 60% the yield is lower than in the reduction at the Hg cathode, where 88% of the dimer were obtained. Possibly higher yields can be obtained with other graphite varieties or other nontoxic cathode materials. But in principle the mercury cathode can be replaced by a graphite cathode.

### Structure of the hydrodimers

All products show for the nitro group characteristic asymmetrical and symmetrical vibrations at 1530–1560 cm^−1^ and 1350–1360 cm^−1^, which, compared to the educts, are shifted to shorter wavelengths.

The hydrodimers show in the upper masses of the mass spectra few fragments and these have a low intensity. The base peak in all hydrodimers results from breaking of the dibenzyl bond and loss of NO_2_ affording the mass = (M^+^/2 − 46).

The C–C bond formation can lead to α,α-, α,β- and ß,ß-coupled products. The ^1^H NMR spectra and MS data support in all cases a ß,ß-coupling. A α,β- or a α,α-coupling would lead to the occurrence of methyl singlets or non-coupled benzylic protons. Such signals were not observed in the spectra of the hydrodimers.

**Scheme 7:**
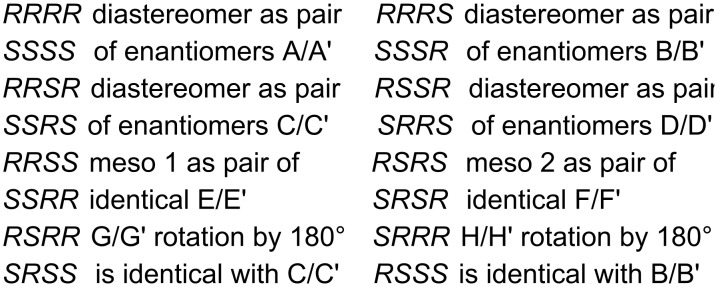
Possible stereoisomers and their mirror images for the hydrodimers **2** and **18–23**; *R* and *S* are the configurations at the stereogenic centers.

The stereochemistry of the hydrodimer results from a β,ß-C–C bond formation and from a α,δ-diprotonation, which creates a dimer with four stereocenters with the exception of dimer **3**, which has only two stereocenters. This means 2^3^ diastereomers can be formed, which are decreased to six diastereomers due to the identity of two pairs of enantiomers as shown in Scheme 7.

Configurations could not be assigned, as data for comparison are not available in the literature and crystals for X-ray diffraction could not be obtained.

The dimers are obtained as mixtures of diastereomers; as they do not differ significantly in their MS and IR spectra the compounds had to be characterized by their ^1^H NMR data. For that purpose the diastereomers were purified as good as possible by flash chromatography and/or HPLC. Single diastereomers are denoted alphanumerically (e.g., **2a**, **2b**). The same letter means for two diastereomers of different hydrodimers that they have similar NMR spectra with regard to chemical shift and multiplicity. This corresponds to a similar rate of elution in chromatography.

### ^1^H NMR spectra

For the hydrodimers **2** and **18**–**23** five different diastereomers **a**–**e** can be identified. In Table 5 the ^1^H NMR data of the five diastereomers **2a**–**e** are assembled.

**Table 5:** δ-Values and multiplicities of the alkyl protons in **2a**–**e**.

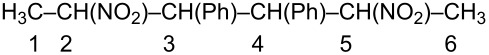						

Isomer	δ (ppm) and multiplicity for H-atom at carbon-atom Nr.:					

	1	6	2	5	3	4

**2a**	1.25, d		4.85, dq		3.44, d	

**2b**	1.28, d	1.90, d	4.91, dq	4.79, dq	3.36 and 3.71, 2 dd	

**2c**	1.74, d		5.21/5.22, 2 dq^a^		3.64, dd	

**2d**	1.29, d		4.47–4.56, m		4.13–4.15, m	

**2e**	1.25, d	1.30, d	4.44, dq	4.62, dq	4.59, dd	3.33, dd

^a^Coupling pattern of the α-nitro protons is verified by NMR-simulation.

The numbering (determining C-1) is arbitrary, but the remaining positions follow unequivocally from the coupling constants (for example in **2b**: *J*_1,2_ = 6.64 Hz, *J*_5,6_ = 6.51 Hz).

**Figure 2:**
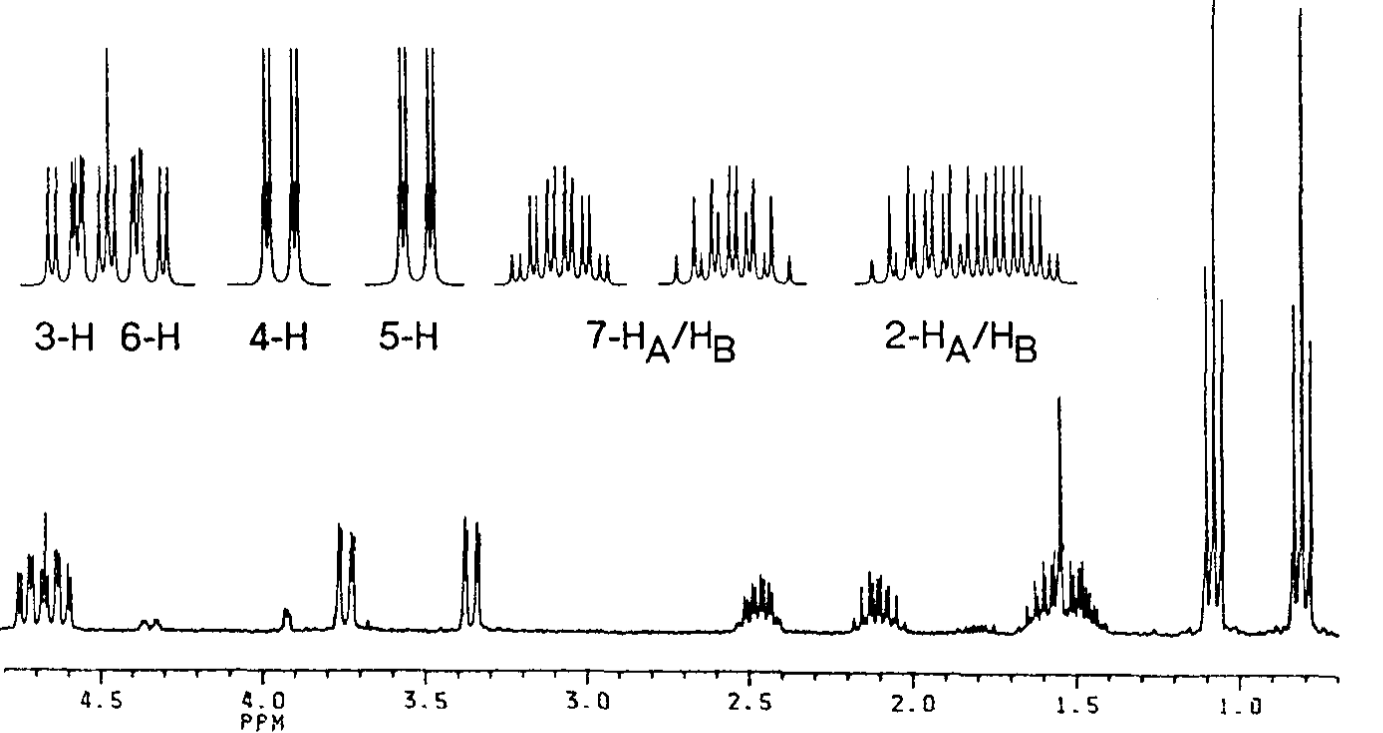
^1^H NMR spectrum of **18b** (without aromatic H); below experimental spectrum, above: simulated signals for 2-H to 7-H.

The diastereomers show remarkable differences in the chemical shifts. This also holds for significant differences in the chemical shifts for formally identical protons in **2b**/**e**. The considerable differences probably result from the anisotropy effect of the aromatic ring and the nitro group. For the nitro group a similar anisotropic cone as for the carbonyl group is assumed [30]. Due to the presence of two nitro and two phenyl groups conformations are possible, where protons are in shielded and unshielded areas. Also phenyl protons appear as broad, high field shifted signals. For **18c** a ratio of high field and normal signals of 4:6 was found. With decoupling experiments they can be clearly assigned to be phenyl protons. Comparable results, as shown for **2**, were found for the mixtures of diastereomers of the other hydrodimers. Besides decoupling experiments also ^1^H NMR simulations give valuable support to assign the complex coupling pattern. This is shown for **18b** where the complexity of the spectrum is strongly increased by the diastereotopic methylene protons (Figure 2).

The ratios of diastereomers for the products **2** and **18**–**23** are summarized in Table 6.

**Table 6:** Ratio^a^ of the diastereomers **a**–**e** from the dimers **2** and **18−23**.

Dimer	**a**	**b**	**c**	**d**	**e**

**2**	10	14	2	1	3
**18**	11	17	4	1	3
**19**	12	20	6	1	6
**20**	7	14	3	1	2
**21**	11	16	3	1	2
**22**	1.7	3.2	1.7	1	5.5
**23**	2	2	Σ 1(for **c**, **d**, **e)**		

^a^Determined by comparing the intensities in the ^1^H NMR spectra of different mixtures; average values from different electrolyses.

The main part of the mixture consists usually of the isomers **a** and **b**. Dimer **22** is an exception being possibly caused by the NH groups of the pyrroles. Their hydrogen bonds could influence the relative energies of the transition states leading to the diastereomers. One of the two *meso*-configurations can be assigned to isomer **22a** because of the missing coupling between the benzylic methine protons.

The ^1^H NMR data obtained for **29**–**31** are compatible with the shown structures.

### ^13^C NMR spectra

The proposed structures were confirmed by their ^13^C NMR spectra. Nearly all diastereomers can be characterized via their ^13^C signals. The regioselective ß,ß-linkage follows clearly from the multiplicities of the carbon atom resonances. Signals of aliphatic quaternary carbon atoms were not detected. The differences between the signals of single diastereomers of a dimer correlate very well with the results of the proton resonance experiments. The measured values agree quite well with increment calculations (Table 7) [31].

**Table 7:** Calculated and experimental ^13^C shifts for **18**.

Carbon atoms	C-1/-8	C-2/-7	C-3/-6	C-4/-5

δ (ppm) calculated	15.0	22.0	90.4	54.4
δ (ppm) found	9.80–10.81	20.98–26.00	88.92–91.70	48.73–51.31

### Elemental analyses

The structures could be secured additionally by elemental analyses and in the case of dimer **20** by high resolution MS. They were obtained from the mixtures of isomers taking into account all elements (C, H, N, halogen).

## Conclusion

The potential controlled cathodic hydrodimerization of 1-nitroalkenes affords a one step electrochemical C–C bond formation to 1,4-dinitro compounds. Applying optimized conditions the hydrodimers are obtained in good to very good yields. Besides mercury also graphite can be used as cathode material. The scope of the reaction is demonstrated in ten nitroalkenes with different 1-aryl and mostly 2-methyl substituents. Likewise the cathodic cyclization of a dinitrodiene could be realized.

The dimerization is chemoselective: the fairly easy reduction of the nitro group can be suppressed and aryl C–Cl and aryl C–F bonds are not cleaved. Additionally a good regioselectivity is obtained, among the possible three coupling products only the ß,ß-linked dimer is found. The diastereoselectivity is moderate, one obtains two main diastereomers (about 70–80% of the mixture of isomers) and one of the six possible diastereomers was not found.

The use of an undivided cell facilitates the electrolysis and lowers the energy consumption. For the preparation of dimer **2** in an undivided cell at cell voltages of 10–15 V, one needs 1.8–2.7 kWh/kg of product, which is much below the 8 kWh/kg, where a technical electrolysis becomes favourable with regard to the energy consumption [32–33].

## Supporting Information

File 1 Experimental procedures, ^1^H, ^13^C NMR and MS spectra and elemental analyses.
