# Supplementary material for: Cathodic hydrodimerization of nitroolefins
Source: Beilstein J Org Chem. 2015 Jul 14;11:1163–74. doi: 10.3762/bjoc.11.131 (PMC4505112; doi:10.3762/bjoc.11.131)
Supplement: File 1 — Experimental procedures, 1H, 13C NMR and MS spectra and elemental analyses. [file Beilstein_J_Org_Chem-11-1163-s001.pdf]

Supporting Information

for

Cathodic hydrodimerization of nitroolefins

Michael Weßling and Hans J. Schäfer*

Address: Organisch-Chemisches Institut der Westfälischen Wilhelms-Universität, Correns-Straße 40, 48149 Münster, Germany

Email: Hans J. Schäfer - schafeh@uni-muenster.de

*Corresponding author

Experimental procedures, ^{1}H, ^{13}C NMR and MS spectra and elemental analyses

Content **S1**

1 Materials and methods **S2**

1.1 Analytical equipment **S2**

1.2 Chemicals **S3**

1.3 Electrochemical equipment **S3**

1.4 Preparative electrolyses S3

2 Preparation of the starting compounds **S5**

2.1 Preparation of the 1-aryl-2-nitroolefins **S5**

2.1.1 General working procedures (GWP) **S5**

2.1.2 Preparation of (*E*)-2-nitro-1-phenylethene (4) (ω-nitrostyrene) **S6**

2.1.3 Preparation of (*E*)-2-nitro-1-phenylprop-1-ene (1) **S6**

2.1.4 Preparation of (*E*)-2-nitro-1-phenylbut-1-ene (8) **S7**

2.1.5 Preparation of (*E*)-(4-methoxyphenyl)-2-nitroprop-1-ene (9) **S7**

2.1.6 Preparation of (*E*)-2-nitro-1-(4-trifluoromethylphenyl)prop-1-ene (10) **S7**

2.1.7 Preparation of (*E*)-(furan-2-yl)-2-nitroprop-1-ene (11) **S8**

2.1.. Preparation of (*E*)-2-nitro-1-(pyrrol-2-yl)prop-1-ene (12) **S8**

2.1.9 Preparation of (E)-1-(2-chlorophenyl)-2-nitropropene (13) **S9**

2.1.10 Preparation of (*E*)-(2,6-dichlorphenyl)-2-nitroprop-1-ene (14) **S9**

2.1.11 Preparation of (*E*)-1-(2,6-difluorophenyl)-2-nitroprop-1-ene (15) **S9**

2.1.12 Preparation of (*E*,*E*)-2,5-dinitro-1,6-diphenylhexa-1,5-diene (16) **S10**

2.1.13 Optimization of the nitroalkene preparation **S10**

3 Cathodic hydrodimerization of nitroalkenes **S11**
3.1 General procedure for the cathodic hydrodimerization **S11**
3.2 Preparative electrolyses **S11**
3.3 Comments to the analysis and comments to the spectroscopic data **S12**
3.4 Cathodic hydrodimerization of 4 to 3 **S12**
3.5 Cathodic hydrodimerization of 1 to 2 **S13**
3.6 Cathodic hydrodimerization of 8 to 18 **S15**
3.7 Cathodic hydrodimerization of 9 to 19 **S16**
3.8 Cathodic hydrodimerization of 10 to 20 **S18**
3.9. Cathodic hydrodimerization of 11 to 21 **S19**
3.10 Cathodic hydrodimerization of 12 to 22 **S21**
3.11 Cathodic hydrodimerization of 13 to 23 **S23**
3.12 Attempted cathodic hydrodimerization of 14 to 26 **S24**
3.13 Cathodic hydrodimerization of 15 to 29 **S26**
3.14 Cathodic hydrodimerization of 16 to 30 **S27**
3.15 Cathodic hydrodimerization of 17 to 31 **S28**
4 References **S29**

1 Materials and methods

1.1 Analytical equipment

IR-Spectra were recorded with a IR-408 spectrometer (Shimadzu) and a IR-421 (Perkin-Elmer). ^{1}H NMR and ^{13}C NMR spectra were measured with a WM300 spectrometer (Bruker) at 300 MHz and 75.5 MHz.- Mass spectra (EI, 70 eV) were obtained with a MAT 312 and a MAT 8230 instrument (Finnigan - MAT). For GC-MS coupling (EI, 70 eV) the gaschromatographs 1400 and 3400 (Varian) and GC-8A (Shimadzu) were combined with mass spectrometers MAT CH7A and MAT 8230 (Finnigan). The following quartz-capillary columns (Macherey & Nagel) were used: column 1: FS-SE-54-CB-0.25 µm, 50 m, 0.32 mm ID, column 2: FS-HP-1-CB-0.25 µm, 50 m, 0.32 mm ID, and column 3: FS-RSL-300-0.3, 3m, 0.32 mm ID. TLC was performed with silicagel 60 F254 (Merck).- For flash chromatography silicagel 60 [40-63 µm] (Merck) was used. HPLC in semi preparative scale was performed with HPLC pump 64 (Knauer), injector Rheodyne 7125 and Knauer column 1: Nucleosil 100-3, 3 µm, 250 mm length, 8 mm ID and column 2: Nucleosil 100-5, 5 µm, 250 mm length, 8 mm ID. Elemental analyses were performed by Microanalytical Laboratory M. Beller, Göttingen.

Melting points are uncorrected. Yields refer to the substance used as minor component. All reactions, where oxygen and water had to be excluded, were done under argon in baked-out glass vessels.

1.2 Chemicals

The employed chemicals were purchased from Aldrich, Merck, Janssen, Fluka and Sigma and were used without further purification, if not stated otherwise. All solvents were purified by distillation and if necessary residual water was removed. The composition of solvents and eluents are given in volume ratios of the components.

1.3 Electrochemical Equipment

Cyclic voltammograms were obtained with a Computer IIe (Apple), a Potentiostat PCA 72 L (Bank Electronic) and the cell EA 876 (Metrohm). Working electrode was an amalgamated gold wire electrode (1 mm diameter, 2 cm length), as counter electrode served a platinum foil (1 cm^2). Reference electrodes were a saturated Kalomel electrode (SCE), +0.27 V vs. NHE and a Marple-electrode.

1.4 Preparative electrolyses

The preparative electrolyses were performed in cells with a cooling jacket and a maximum content of 150 mL. The cells are shown in Figure S1 (Hg cathode) and Figure S2 (graphite cathode). The geometrical surface of the Hg-cathode was 19 cm^2. As graphite electrographite P127 SGL Carbon was used. Current sources were the potentiostat HP 88 and St 88 Fa. Bank Electronic, the current consumption was measured with a digital DC-integrator.

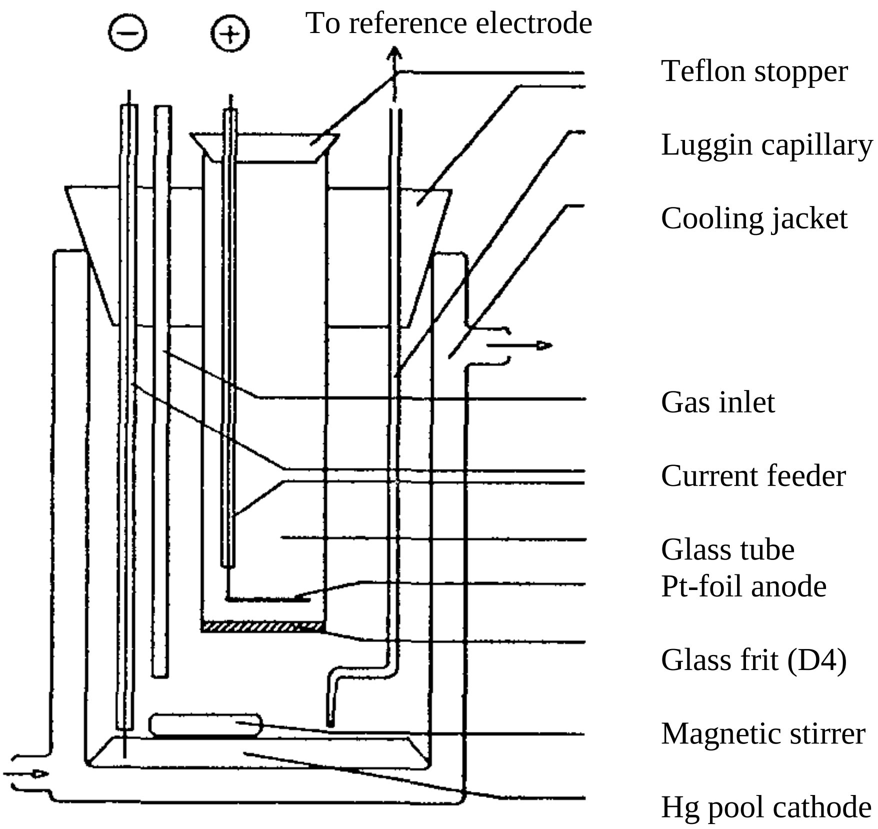

Figure S1: Beaker-type cell with a Hg-cathode; divided cell with glass frit, undivided cell without glass frit.

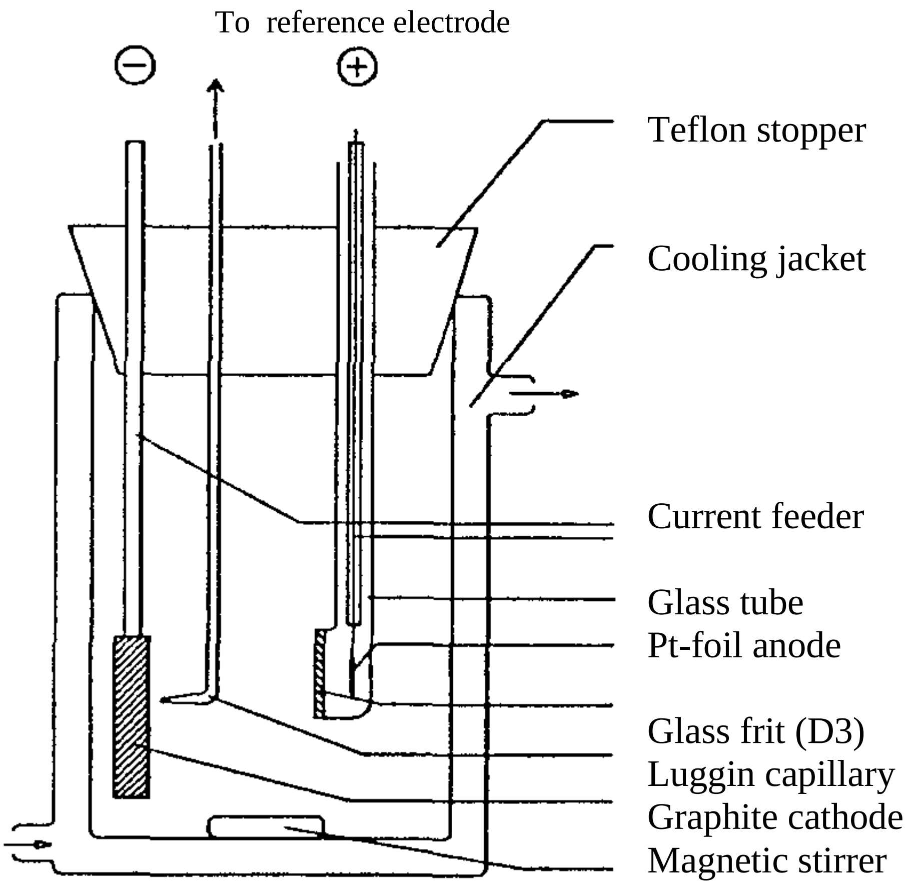

Figure S2: Beaker type cell with a graphite cathode; divided cell with glass frit, undivided cell without glass frit

2 Preparation of the starting compounds

2.1 Preparation of the 1-aryl-2-nitroolefins

2.1.1 General working procedures (GWP)

The GWP, given here, is addressed in the specific product information. Specific product information is placed under the corresponding substrates.

2.1.1.1 GWP 1 [1]

Freshly distilled aldehyde (0.1 mol) and the nitroalkane (0.1 mol) are dissolved in absolute ethanol (20 mL) containing *n*-butylamine (2 mL) as catalyst and are refluxed for the given time. The initially colourless solution changes depending on the educts with advancing reaction from yellow to orange and red to partially brown or black. The coloured reaction mixture is slowly cooled to rt and then to 0 °C. Thereby in favourable cases the nitroolefin crystallizes and can be filtered off with suction. By concentrating the mother liquor further

crystal precipitations can be obtained. Recrystallization of the crude product from the given solvent leads to pure nitrostyrene derivatives.

If no crystallization occurs after long standing in the refrigerator, the alcohol is rotaevaporated and the remaining oil dissolved in diethyl ether (100 mL) and the ethereal solution is washed successively with dil. HCl, water, a sat. $NaHCO_3$-solution and water. After drying ($MgSO_4$) and rotaevaporating of diethyl ether an oily residue is obtained, which can be crystallized by cooling and rubbing with a small amount of petroleum ether or dissolving/crystallization from petroleum ether/diethyl ether.

2.1.1.2 GWP 2 [2]

Freshly distilled aldehyde (0.2 mol) and the nitroalkane (0.4 mol) and ammonium acetate (10 g) are dissolved in acetic acid (100 mL) and refluxed for 2 h. The deeply coloured reaction solution is stirred in ice water (100 mL). If the nitroolefin precipitates it is filtered off with suction and recrystallized from the given solvent. If no crystals are obtained, the oil is dissolved in diethyl ether (100 mL), the organic phase is washed with an aqueous sat. $NaHCO_3$-solution (3 × 30 mL) and with water, is then dried ($MgSO_4$) and the solvent is rotaevaporated. The partially crystalline residue is recrystallized from the given solvent.

2.1.2 Preparation of (*E*)-2-nitro-1-phenylethene (4) (ω-nitrostyrene)

Yellow needles (preparation [3]), Mp. 57-58 °C ([3,5] 58 °C).- ^{1}H NMR ($CDCl_3$): δ (ppm) = 7.42-7.55 (m, 5 H, aromat. H), 7.59 (d, *J* = 13.6 Hz, 1 H, $CH=CHNO_2$), 8.02 (d, *J* = 13.6 Hz, 1 H, $CH=CHNO_2$).

2.1.3 Preparation of (*E*)-2-nitro-1-phenylprop-1-ene (1)

Benzaldehyde (10.61 g, 0.1 mol, **6a**) and nitroethane (7.51 g, 0.1 mol, **7b**) afford using GWP 1 after recrystallization from abs. ethanol yellow crystalline **1** (8.85 g, 54 mmol, 54%).- Mp.: 64-65 °C (Lit. [1,6] 65 °C).- IR (KBr): ν (cm^{-1}) = 3030 (=CH), 2940 (CH), 1640 (C=C), 1505 (NO_2), 1425, 1380, 1310 (NO_2), 1205, 960, 930, 855, 750, 680 (=CH).-^{1}H NMR ($CDCl_3$): δ (ppm) = 2.46 (s, 3 H, CH_3), 7.35-7.51 (m, 5 H, aromat. H), 8.10 (s, 1 H, $CH=CNO_2$).

MS (GC/MS-coupling, EI, 70 eV): m/z (%) = 163 (38) [M^+], 146 (14) [M^+-OH], 135 (9) [M^+-CO], 133 (3) [M^+-NO], 118 (5), 117 (19) [M^+-NO_2], 116 (33) [M^+-HNO_2], 115 (100) [M^+-H_2NO_2], 105 (35) [M^+-CO-NO], 91 (34) [C_7H_7], 77 (12), 65 (10), 57.5 (15) [base peak $^{2+}$], 51 (12), 39 (12).

2.1.4 Preparation of (*E*)-2-nitro-1-phenylbut-1-ene (8)

10.61 g (0.1 mol) **6a** and 8.91 g (0.1 mol) 1-nitropropane (**7c**) afford with GWP 1 after vacuum distillation **8** (10.97 g, 62 mmol, 62%) as yellow liquid. B.p. 72-77 °C/0.3 mbar (125-129 °C/10 Torr (13.3 mbar)[1,7]). n^{25}_D = 1.5812 (n^{25}_D = 1.5832). -IR (film): ν (cm^{-1}) = 3030 (=CH), 2950, 2910, 2850 (CH), 1645 (C=C), 1510 (NO_2), 1440, 1320 (NO_2), 1205, 1010, 935, 855, 795, 750, 685 (=CH).-^{1}H NMR ($CDCl_3$): δ (ppm) = 1.28 (t, *J* = 7.39 Hz, 3 H, CH_3), 2.87 (q, *J* = 7.39 Hz, 2 H, CH_2), 7.37- 7.51 (m, 5 H, aromat. H), 8.02 (s, 1 H, $CH=CNO_2$).- MS (GC/MS-coupling, EI, 70 eV): m/z (%) = 177 (14) [M^+], 160 (12) [M^+-OH], 149 (8) [M^+-CO], 131 (11) [M^+-NO_2], 130 (11) [M^+-HNO_2], 129 (26) [M^+-H_2NO_2], 116 (28), 115 (48) [M^+-HNO_2-CH_3], 91 (100) [$C_7H_7^+$], 77 (21), 65 (13), 51 (16).

2.1.5 Preparation of (*E*)-(4-methoxyphenyl)-2-nitroprop-1-ene (9)

Using GWP 2, 4-methoxybenzaldehyde (27.23 g, 0.2 mol, **6b**) and **7b** (30.03 g, 0.4 mol) afford after recrystallization from abs. EtOH bright yellow crystals of **9** (16.98 g, 88 mmol, 44%).- Mp.**:** 43-44 °C ([2] 44-45 °C, [6] 48 °C).- IR (KBr): v (cm^{-1}) = 3025 (=CH), 2960, 2920 (CH), 2820 (OCH_3), 1640, 1600 (C=C), 1510, 1490 (NO_2), 1450, 1385, 1295 (NO_2), 1250 (C-O-C), 1165, 1020, 975, 865, 810 (=CH, 1,4-disubstitution).-^{1}H NMR ($CDCI_3$): δ (ppm) = 2.47 (s, 3 H, CH_3), 3.86 (s, 3 H, OCH_3), 6.94-7.03 (m, 2 H, aromat. 3-, 5-H), 7.39-7.48 (m, 2 H, aromat. 2-, 6-H), 8.07 (s, 1 H, $CH=CNO_2$).- MS (GC/MS-coupling, EI, 70 eV): *m/z* (%) = 193 (100) [M^+], 176 (2) [M^+-OH], 162 (4) [M^+-OMe], 146 (84) [M^+-HNO_2], 131 (30) [M^+-HNO_2-CH_3], 115 (34) [M^+-HNO_2-OMe], 103 (26) [M^+-HNO_2-CO-CH_3], 91 (22) [C_7H_7], 77 (24), 63 (11), 51 (14).

2.1.6 Preparation of (*E*)-2-nitro-1-(4-trifluoromethylphenyl)prop-1-ene (10)

As **10** cannot be prepared in acceptable yields neither according to GWP 1 nor according to GWP 2 a procedure according to [4] was used. Aldehyde **6c** (17.41 g, 0.1 mol) and n-butylamine (7.32 g, 0.1 mol) in toluene (25 ml) were refluxed in a water trap until no longer water separated. The toluene was rotaevaporated and to the residue nitroethane (7.51 g, 0.1 mol, **7b**) and acetic acid (25 ml) were added and the colourless mixture was refluxed for 40 min. The deep red solution was poured under strong stirring on ice (140 g) and the dark crystal pap was sucked off. Recrystallization from abs. ethanol affords light yellow, fine crystalline **10** (9.74 g, 42 mmol, 42%).-

Mp.: 37-38 °C ([6] 37-39 °C).- IR (KBr):ν (cm^{-1}) = 3050 (=CH), 2980, 2930 (CH), 1660, 1610 (C=C), 1520, 1505 (NO_2), 1410, 1315 (NO_2), 1155 , 1110, 1060, 970, 840 (=CH, 1,4-

disubstitution).- ^{1}H NMR ($CDCl_3$): δ (ppm) = 2.45 (s, 3 H, CH_3), 7.57 (d, *J* = 8.3 Hz, 2 H, aromat. 3-, 5-H), 7.72 (d, *J* = 8.3 Hz, 2 H, aromat. 2-, 6-H), 8.08 (s, 1 H, $CH{=}CNO_2$).- MS (GC/MS-coupling, EI, 70 eV): *m/z* (%) = 231 (34) [M^+], 214 (19) [M^+-OH], 212 (8) [M^+-F], 203 (12) [M^+-CO], 201 (8) [M^+-NO], 185 (18) [M^+-NO_2], 184 (26) [M^+-HNO_2], 173 (36) [M^+-CO-NO], 165 (68) [M^+-HNO_2-F], 164 (34), 145 (27) [M^+-OH-CF_3], 134 (33) [M^+-CO-CF_3], 133 (29), 116 (59) [M^+-NO_2-CF_3], 115 (100) [M^+-HNO_2-CF_3], 63 (10), 42 (13), 39 (18).

2.1.7 Preparation of (*E*)-(furan-2-yl)-2-nitroprop-1-ene (11)

Furan-2-carbaldehyde (25.94 g, 0.27 mol, **6d**) und nitroalkane **7b** (20.27 g, 0.27 mol) afford according to GWP 1 after recrystallization from abs. EtOH the nitroalkene **11** (30.85 g, 202 mmol, 75%) in bright yellow crystals. Mp.**:** 47-48 °C (Lit.[1] 48-49 °C).-

IR (KBr): *v* (cm^{-1}) = 3100 (=CH), 2920 (CH), 1635, 1615 (C=C), 1495 (NO_2), 1380, 1295 (NO_2), 1010, 970, 910, 850, 755.-^{1}H NMR ($CDCl_3$): δ (ppm) = 2.59 (s, 3 H, CH_3), 6.59 (dd, *J* = 1.8, 3.5 Hz, 1 H, O-CH=C*H*-CH), 6.83 (d, *J* = 3.5 Hz, 1 H, O-CH=CH-C*H*), 7.65 (d, *J*= 1.8 Hz, 1 H, O-C*H*=CH-CH), 7.86 (s, 1 H, $CH{=}CNO_2$).

MS (GC/MS-coupling, EI, 70 eV): *m/z* (%) = 153 (30) [M^+], 136 (4) [M^+-OH], 106 (9) [M^+-HNO_2], 95 (15) [M^+-CO-NO], 83 (100), 78 (41), 77 (85), 67 (8) [$C_4H_3O^+$], 55 (30), 53 (40), 51 (55), 44 (46), 41 (49), 40 (71).

2.1.8 Preparation of (*E*)-2-nitro-1-(pyrrol-2-yl)-prop-1-ene (12)

According to GWP 1 pyrrol-2-carbaldehyde (25 g, 0.26 mol, **6e**) is reacted with nitroalkane **7b** (19.52 g, 0.26 mol). The obtained red-black oil did not form crystals. Therefore it was dissolved in diethyl ether, cleared with charcoal and the suspension filtered over a 6 cm silicagel column with petroleum ether/diethyl ether, 4 : 1). A red viscous liquid (17.4 g) is obtained, which besides **12** contains a great amount of **6e**. Multiple recrystallizations from cyclohexane afford **12** (4.05 g, 2.7 mmol, 10%). Mp.: 83-85 °C.- IR (KBr): *v*(cm^{-1}) = 3400, 3350 (NH), 3100 (=CH), 2900 (CH), 1630 (C=C), 1485 (NO_2), 1425, 1355, 1260 (NO_2), 1115, 1040, 995, 940, 850, 730.- ^{1}H NMR ($CDCl_3$): δ(ppm) = 2.51 (s, 3 H, CH_3), 6.41-6.48 (m, 1 H, ring-H), 6.71-6.78 (m, 1 H, ring- H), 7.10-7.17 (m, 1 H, ring-H), 8.18 (s, 1 H, $CH{=}CNO_2$), 8.75-9.10 (br s, 1 H, NH).- MS (direct inlet, EI, 70 eV): *m/z* (%) = 153 (20) [M^++H], 152 (62) [M^+], 135 (5) [M^+-OH], 119 (10), 106 (30) [M^+-NO_2], 105 (65) [M^+-HNO_2], 104 (69) [M^+-H_2NO_2], 96 (58), 95 (100) [M^++H-CO-NO], 94 (64) [M^+-CO-NO], 91 (38), 80 (66) [$C_5H_6N^+$], 79 (75), 66 (66) [$C_4H_4N^+$], 52 (70), 51 (69).- $C_7H_8N_2O_2$ (152.2) Calc. C 55.26, H 5.30, N 18.41; Found C 55.13, H 5.39, N 18.46.

2.1.9 Preparation of (*E*)-1-(2-chlorophenyl)-2-nitropropene (13)

2-Chlorobenzaldehyde (42.17 g, 0.3 mol, **6f**) is reacted according to GWP 1 with **7b** (22.52 g). As the crystallization fails to appear, unreacted aldehyde was removed by way of the bisulfite adduct. The obtained oily crude product could be crystallized by cooling in a dry ice/methanol bath by rubbing with petroleum ether. Sucking off the crystals and thoroughly washing with ice cold EtOH affords bright yellow crystals of **13** (34.39 g, 174 mmol, 58 %).- Mp.**:** 40-41 °*C* (Lit.[2] 44-45 °C).- IR (KBr): *v* (cm^{-1}) = 3075 (=CH), 2940, 2830 (CH), 1645, 1590 (C=C), 1510 (NO_2), 1465, 1430, 1385, 1320 (NO_2), 1030 (C-Cl), 865, 765 (=CH, 1,2-disubstitution).-^{1}H NMR ($CDCl_3$): δ(ppm): 2.35 (s, 3 H, CH_3), 7.30-7.41 (m, 3 H, aromat. H), 7.44-7.52 (m, 1 H, aromat. H), 8.19 (s, 1 H, $CH{=}CNO_2$).- MS (GC/MS-coupling, EI, 70 eV): *m/z* (%) = 199/197 (6/18) [M^+], 162 (52) [M^+-Cl], 152/150 (5/15) [M^+- HNO_2], 141/139 (2/8) [M^+-CO-NO], 132 (64) [M^+-CI-NO], 116 (58) [M^+-Cl-NO_2], 115 (100) [M^+-HNO_2-Cl], 103 (11), 89 (19), 63 (16), 57.5 (19) [base peak^{2+}], 39 (19).

2.1.10 Preparation of (*E*)-(2,6-dichlorophenyl)-2-nitroprop-1-ene (14)

Using GWP 2 the conversion of 2,6-dichlorobenzaldehyde (**6g,** 6.0 g, 34 mmol) and **7b** (5.25 g, 70 mmol) affords after recrystallization from cyclohexane yellow crystalline **14** (2.81 g, 12 mmol, 36 %).- Mp. 49-50 °C (Lit.[4] 48-49 °C).- IR (KBr): *v* (cm^{-1}) = 3010 (=CH), 2950, 2900 (CH), 1575, 1550 (C=C), 1510 (NO_2), 1420, 1380, 1325, 1310 (NO_2), 1080 (C-Cl), 975, 765 (=CH, 1,2,3-trisubstitution).-^{1}H NMR ($CDCl_3$) [4]: δ (ppm) = 2.13 (s, 3 H, CH_3), 7.27-7.47 (m, 3 H, aromat. H), 7.86 (s, 1 H, $CH{=}CNO_2$).- MS (GC/MS-coupling, EI, 70 eV): *m/z* (%) = 235/233/231 (2/11/16) [M^+], 198/196 (21/98) [M^+-Cl], 188/ 186/184 (4/30/30) [M^+-HNO_2], 168/166 (37/100) [M^+-NO-Cl], 151/149 (31/81) [M^+-HNO_2-Cl], 115 (85) [M^+-NO_2-2 Cl], 87 (24), 75 (18).

2.1.11 Preparation of (*E*)-1-(2,6-difluorophenyl)-2-nitroprop-1-ene (15)

Using GWP 1 2,6-difluorobenzaldehyde (**6h,** 5.0 g, 35 mmol) and **7b** (2.63 g, 35 mmol) yield after recrystallization yellow crystals of **15** (4.30 g, 22 mmol, 62 %).- Mp.: 63-64 °C.- IR (KBr): *v* (cm^{-1}) = 3050 (=CH), 2970 (CH), 1665, 1610, 1580 (C=C), 1510 (NO_2), 1450, 1385, 1305 (NO_2), 1200 (C-F), 1095, 970, 775 (=CH, 1,2,3-trisubstitution).- ^{1}H NMR ($CDCl_3$):δ (ppm): = 2.23 (m, 3 H, CH_3), 6.95-7.05 (m, 2 H, aromat. 3-, 5-H), 7.36-7.47 (m, 1 H, aromat. 4-H), 7.85 (s, 1 H, $CH{=}CNO_2$).- MS (GC/MS-coupling, EI, 70 eV): *m/z* (%) = 199 (55) [M^+], 153 (40) [M^+-NO_2], 152 (63) [M^+-HNO_2], 151 (59) [M^+-H_2NO_2], 141 (13) [M^+-NO-CO], 133 (51) [M^+-HNO_2-F], 127 (100) [$C_7H_5F_2^+$], 114 (11) [M^+-HNO_2-2 F], 107 (12), 101 (16)

[$C_5H_3F_2^+$].- $C_9H_7F_2NO_2$ (199.2): Calc. C 54.28, H 3.54, F 19.1, N 7.03; Found C 54.43, H 3.64, F 19.2, N 7.02.

2.1.12 Preparation of (*E,E*)-2,5-dinitro-1,6-diphenylhexa-1,5-diene (16)

2.1.12.1. Preparation of 1.4-Dinitrobutane: According to Lit. [8] from 1,4-dibromobutane (103.6 g, 0.48 mol) fine crystalline 1.4-dinitrobutane (9.95 g, 67 mmol, 14%) is obtained.- Mp.: 31-32 °C (Lit.[6] 33.5-34.5 °C).- IR (KBr): *v* (cm^{-1}) = 2950, 2920 (CH), 1545 (NO_2), 1465, 1380, 1355 (NO_2), 1295, 1180.- ^{1}H NMR ($CDCl_3$): δ(ppm) = 2.08-2.20 (m, 4 H, 2-, 3-H), 4.40-4.53 (m, 4 H, 1-, 4-H).- MS (GC/MS-coupling, EI, 70 eV): *m/z* (%) = 148 (0) [M^+], 102 (0.5) [M^+-NO_2], 74 (2.5) [M^+/2], 55 (100) [M^+-HNO_2-NO_2], 41 (22), 39 (21).

2.1.12.2. Preparation of (*E,E*)-2,5-dinitro-1.6-diphenylhexa-1.5-diene (**16**) [9]

1a (6.45 g, 61 mmol) is added to a solution of 1,4-dinitrobutane (4.50 g, 30.5 mmol) in abs. EtOH (30 mL), it is warmed until to complete dissolution and 1,2-diaminoethane (0.5 mL) is added. The mixture is stirred for 6 d at rt. The ochreous coloured precipitate is sucked off. Recristallization from acetone affords **16** as bright yellow crystal plates (5.75 g, 18 mmol, 59%).- Mp.: 174-175 °C (Lit.[9] 174 °C).- IR (KBr): *v* (cm^{-1}) = 3070 (=CH), 2950 (CH), 1645, 1600 (C=C), 1510 (NO_2), 1450, 1295 (NO_2), 1225, 1010, 940, 870, 760, 675 (=CH).-^{1}H NMR ($CDCl_3$): δ (ppm) = 3.24 (s, 4 H, 2 CH_2), 7.40-7.63 (m, 10 H, aromat. H), 8.20 (s, 2 H, 2 CH=CNO_2).- MS (direct inlet, EI, 70 eV): *m/z* (%) = 324 (0) [M^+], 307 (5) [M^+-OH], 278 (10) [M^+-NO_2], 261 (15) [M^+-NO_2-OH], 248 (14) [M^+-NO_2-NO], 215 (19), 162 (18) [M^+/2], 145 (20), 131 (25), 116 (100) [M^+/2-NO_2], 115 (89) [M^+/2-HNO_2], 105 (39), 91 (52), 77 (51), 51 (37).

2.1.13 Optimization of the nitroalkene preparation

GWP 1 is supplemented as follows: In the case that no crystals are obtained either spontaneously or after long cooling, one dissolves in diethyl ether the oily product mixture obtained after rotaevaporation of the alcohol. The ethereal solution is washed with diluted HCl, $NaHCO_3$-solution and water. Then the organic layer is shaken for 30 min with a saturated aqueous $NaHSO_3$ solution. The aqueous phase and the bisulfite adduct are removed, the ether phase is washed with water, dried ($MgSO_4$) and the diethyl ether rotaevaporated. The residue affords after recrystallization pure, crystalline nitroalkenes.

3 Cathodic hydrodimerization of nitroalkenes

3.1General procedure for the cathodic hydrodimerization

3.2 Preparative electrolyses

3.2.1 Electrolyses

The cathodic hydrodimerization is performed based on the results of the experiments shown in Tables 1 and 2 (Theoretical part) in an undivided cell (Figure 1a without diaphragm) at a mercury pool cathode at T = 0 °C (outside cooling). Electrolyte was a 0.2 M solution of tetraethylammonium *p*-toluenesulfonate (TEA-*p*Tos) in DMF. The supporting electrolyte contained 2 % water, is hygroscopic and was not dried. DMF is also not dried. To remove free amines the solvent was purged for some time with dry nitrogen gas. The supporting electrolyte is purged before the electrolyis for 10 min with nitrogen to remove dissolved oxygen. The given amount of substrate dissolved in 20 mL supporting electrolyte is given into the cell. The Luggin capillary is filled with electrolyte and positioned close to the cathode. Subsequently the anode is placed in the cell with a distance of 1 cm to the mercury surface. It is secured that in spite of the small distance to the cathode stirring of the mercury pool (500 t/min) can be done without disturbances of the current flow. Under argon the solution of the olefin is electrolyzed at the given potential until the current has decreased to 1–10 mA (i_{start} = 250–450 mA). During the reduction – especially when stirring is stopped – at the cathode a deep colouring of the solution being red, brown or violet depending on the olefin can be observed. It diffuses into the solution and is discoloured at the anode. In the course of the electrolysis this leads to the colouring of the electrolyte.

3.2.2 Work-up

The coloured electrolyte is separated from the mercury and then a saturated NaCl solution (80 mL) is added, which leads to a lighter colour and the precipitation of NaCl. The suspension is extracted with petroleum ether/diethyl ether (2:1) (3 × 50 mL), the combined extracts are washed with water (2 × 30 mL), dried ($MgSO_4$) and the solvent rotaevaporated. The final purification by flash chromatography with petroleum ether/dichloromethane (1:1) leads to pure hydrodimers as colourless, partially crystalline mixtures of diastereomers, whose further separation is specified below.

3.3 Comments to the analysis and comments to the spectroscopic data

GC analysis (column 1, 2) of the mixtures of diastereomers especially for quantification of the diastereomer ratios is only limited possible in other cases impossible at all, as the hydrodimers decompose partially or totally in the injector or on the GC-column. With a short GC-column

(column 3: 50 °C, 10 °C/min, 330 °C) with shorter retention times the dimers can be detected, however, the separation is insufficient. But, the absence of lower molecular weight compounds or oligomers can be verified in all cases.

The ^{1}H NMR spectra of the mixtures are due to overlapping signals generally very complex. In order to assign for each component one of the possible diastereomers and make possible a semiquantitative estimation of the diastereomer ratios the following procedure was applied: The mixtures in most cases can be separated by flash chromatography and/or HPLC in fractions, which contain different diasteomers as main component. ^{1}H and ^{13}C NMR-spectroscopic investigations of the fractions enable via the different intensities and coupling constants an assignment of the signals to single diastereomers and this way to a structure. In most cases up to five diastereomers per hydrodimer could be identified. Additionally this procedure allows estimating the isomer ratios. The assignment of a configuration is not possible. Therefore the diastereomer is named alphanumerically (compound. Nr. + small letter) in the order it is eluted from the flash- or HPLC column. Thereby diastereomers of different hydrodimers but similar diastereomers get usually the same letters. In the IR-spectra practically no differences, in the mass spectra only slight differences between the isomers with regard to intensities and fragment ions were found.

3.4 Cathodic hydrodimerization of 4 to 3

Using the general working procedure (GWP in chapt. 3.1) (*E*)-2-nitro-1-phenylethene (**4**) [1.29 g, 8.65 mmol] were electrolyzed at a potential of −0.9 to −1.5 V until 875 C (1.05 F mol^{-1}) were consumed. Directly after applying the working potential the product **3** precipitates as colourless solid. For that reason altogether 35 ml electrolyte were used to maintain the stirring of the suspension. The precipitate also causes problems in the potential control because it deposited at the Luggin capillary, which led to fluctuations in the potential and the current. For that reason the potential was changed up to −1.5 V to guarantee a sufficient current in the electrolysis. Deviating from the GWP the work-up was changed: The suspension was separated from the mercury, then water was added and the precipitate was collected by filtration with suction, digested with much ethanol and dried at the vacuum of the oil pump.

Presumably the trimer of (*E*)-2-nitro-1-phenyethene (**4**) is obtained as major product (**3**, 0.93 g, 3.09 mmol, 71%) in colourless crystals. **3** is in all usual solvents practically insoluble. For that reasons NMR-investigations are not possible.

In the EI-mass spectrum of the solid heated to 300 °C the most pronounced peak was *m/z* = 149, which is the mass of nitroolefin **4**. There were also groups with small peaks up to *m/z* = 460, these, however, allowed no assignment to a structure. In a LDI-mass spectrum of the product the most intensive peak was *m/z* = 434. This mass possibly can be assigned to a trimer: 3 × 149 +2 H +H^+ - O.

Mp.: 238-242 °C (dec.), ref.[10] 238 °C (dec.) for a compound assigned to 1,4-dinitro-2,3-diphenylbutane.- IR (KBr): ν (cm^{-1}) = 3050, 3025 (CH), 2900 (CH), 1610 (C=C), 1555 (NO_2), 1540, 1490, 1450, 1360 (NO_2), 1335, 1300, 1275, 1220, 1195, 1155, 1125, 1085, 1075, 1025, 915, 850, 815, 730, 690 (=CH).- Further spectroscopic data cannot be obtained due to the aforementioned reasons.- $C_{16}H_{16}N_2O_4$ (300.3): Calc. C 63.99, H 5.37, N 9.33; Found: C 63.86, H 5.25,N 9.25. The trimer leads to the same C,H,N-analysis.

3.5 Cathodic hydrodimerization of 1 to 2

3.5.1. Hydrodimerization at a mercury cathode

Using the GWP (chapt. 3.1) (*E*)-2-nitro-1-phenylprop-1-ene (**1**, 0.82 g, 5.03 mmol) are electrolyzed at −0.95 V until 476 C (0.98 F mol^{-1}) are consumed. 2,5-Dinitro-3,4-diphenylhexane (**2**, 0.73 g, 2.22 mmol, 88%) are obtained as colourless oily and partially crystalline mixture of diastereomers. Flash chromatography with petroleum ether/acetone (8:1) allows to identify five diastereomers in a ratio **2a**:**b**:**c**:**d**:**e** = 10:14:2:1:3 (^{1}H NMR).

Mp.: **2a**: 138-142 °C (dec.), **2e**: 182-186 °C (dec.).

IR (KBr): ν(cm^{-1}) = 3035, 3020 (=CH), 2950, 2895 (CH), 1610 (C=C), 1550 (NO_2), 1505, 1460, 1390, 1360 (NO_2), 1305, 1095, 1085, 870, 785, 710, 695 (=CH), 660.-

^{1}H NMR ($CDCl_3$):

2a: δ (ppm) = 1.25 (d, $J_{1,2}$ = $J_{5,6}$ = 6.6 Hz, 6 H, 2 CH_3), 3.44 (d, $J_{2,3}$ = $J_{4,5}$ =11.0 Hz, 2 H, 3-, 4-H), 4.85 (dq, $J_{1,2}$ = $J_{5,6}$ = 6.6 Hz, $J_{2,3}$ = $J_{4,5}$ = 11.0 Hz, 2 H, 2-, 5-H), 7.2-7.4 (m, 10 H, aromat. H).

2b: δ (ppm) = 1.28 (d, $J_{1,2}$ = 6.64 Hz, 3 H, diastereotopic 1-CH_3), 1.90 (d, $J_{5,6}$ = 6.51 Hz, 3 H, diastereotopic 6-CH_3), 3.36, 3.71 (2dd, $J_{2,3}$ = $J_{4,5}$ = 11.19 Hz, $J_{3,4}$ = 2.65 Hz, 2 H, diastereotopic 3-, 4-H), 4.79 (dq, $J_{4,5}$ = 11.19 Hz, $J_{5,6}$ = 6.51 Hz, 1 H, diastereotopic 5-H), 4.91 (dq, $J_{1,2}$ = 6.64 Hz, $J_{2,3}$= 11.19 Hz, 1H, diastereotopic 2-H), 6.5-6.8, 7.1-7.35 (m, 10 H, aromat. H).

2c: δ (ppm) = 1.74 (d, $J_{1,2}$ = $J_{5,6}$ = 6.51 Hz, 6 H, 2 CH_3), 3.64 (dd, $J_{2,3}$ = $J_{4,5}$ = 5.37 Hz, $J_{3,4}$ = 1.83 Hz, 2H, 3-, 4-H), 5.21, 5.22 (two incomplete resolved dq, $J_{1,2}$ = $J_{5,6}$ = 6.51 Hz, $J_{2,3}$ = $J_{4,5}$ = 5.37 Hz, 2H, diastereotopic 2-, 5-H), 6.5-6.8, 7.1 -7.35 (m, 10 H, aromat. H).

The coupling pattern of the α-nitroprotons is verified by NMR-simulation.

2d: δ (ppm) = 1.29 (d, $J_{1,2} = J_{5,6}$ = 6.76 Hz, 6 H, 2 CH_3), 4.13-4.15 (m, 2 H, 3-,4-H), 4.47-4.56 (m, 2 H, 2-, 5-H), 7.3-7.5 (m, 10 H, aromat. H).

2e: δ (ppm) = 1.25 (d, $J_{1,2}$ = 6.75 Hz, 3 H, diastereotopic 1-CH_3), 1.30 (d, $J_{5,6}$ = 6.79 Hz, 3 H, diastereotopic 6-CH_3), 3.33 (dd, $J_{3,4}$ = 12.35 Hz, $J_{4,5}$ = 3.00 Hz, 1 H, diastereotopic 4-H), 4.44 (dq, $J_{1,2}$ = 6.75 Hz, $J_{2,3}$ = 4.37 Hz, 1 H, diastereotopic 2-H), 4.59 (dd, $J_{2,3}$ = 4.37 Hz, $J_{3,4}$ =12.25 Hz, 1 H, diastereotopic 3-H), 4.62 (dq, $J_{4,5}$ = 3.00 Hz, $J_{5,6}$ = 6.79 Hz, 1 H, diastereotopic 5-H), 7.2-7.5 (m, 10 H, aromat. H).

^{13}C NMR ($CDCl_3$)*:*

2a: δ (ppm) = 18.81 (q, C-1/-6), 50.75 (d, C-3/-4), 84.81 (d, C-2/-5), 128.12, 128.20 (2 d, aromat. C-2/-3/-5/-6), 130.60 (d, aromat. C-4), 132.96 (s, aromat. H, C-1).

2b: δ (ppm) = 19.03, 19.06 (2 q, C-1/-6), 49.47, 50.91 (2 d, C-3/-4), 84.55, 84.93 (2 d, C-2/-5), 127.91, 128.11, 128.35, 128.40, (4 d, aromat. C-2/-3/-5/-6), 130.01 (d, aromatic. C-4), 133.08, 133.70 (2 s, aromat. C-1).

2c + 2d: δ (ppm) = 11.62, 18.36 (2 q, **d**-, **c**-C-1/-6), 50.55, 53.39 (2 d, **c**-, **d**- C-3/-4), 83.44, 83.60 (2 d, **c**-, **d**-C-2/-5), 128.18, 128.87, 129.26, 129.76 (d), 134.10 (s).

2e (CD_2Cl_2): δ (ppm) = 12.21, 17.64 (2 q, C-1/-6), 50.31, 52.22 (2 d, C-3/-4), 83.19, 84.53 (2 d, C-2/-5), 128.92, 129.27, 129.35, 129.66, 129.88 (d), 134.33, 135.08 (2 s, aromat. C-1).

MS (GC/MS-coupling, EI, 70 eV): m/z (%) = 328 (1.2) [M^+], 312 (0.2) [M^+-O], 281 (0.6) [M^+-HNO_2], 265 (1.2) [M^+-HNO_2-O], 251 (1) [M^+-HNO-NO], 235 (2.3) [M^+-HNO_2-NO_2], 208 (3.9) [M^+-HNO_2-H_2C=$CHNO_2$, McLafferty], 207 (3.7) [M^+-HNO_2 - H_3CCHNO_2, Benzyl-cleav.], 164 (4) [M^+/2], 119 (23), 118 (100) [$C_6H_5C_3H_5^+$], 117 (47), 115 (17), 105 (28), 91 (22) [$C_7H_7^+$], 77 (6), 65 (4).

$C_{18}H_{20}N_2O_4$ (328.4): calc. C 65.84, H 6.14, N 8.53; found: C 65.97, H 6.19, N 8.49.

3.5.2 Hydrodimerization of **1** to **2** at a graphite cathode

Using the GWP (chapt. 3.1) (*E*)-2-nitro-1-phenylprop-1-ene (**1**, 1.00 g, 6.13 mmol) was electrolyzed at −1.3 V until 826 C (1.40 F mol^{-1}) were consumed. Deviating from the GWP, in this case at a graphite cathode in an undivided cell (Figure 1b without diaphragm) was electrolyzed in 25 mL of electrolyte to afford 2,5-dinitro-3,4-diphenylhexane (**2**, 0.60 g, 1.83 mmol, 60%) as colourless partially crystalline mixture of diastereomers.

3.6 Cathodic hydrodimerization of 8 to 18

According to the GWP (chapt. 3.1) 2-nitro-1-phenylbut-1-ene (**8**, 0.99 g, 5.59 mmol) was electrolyzed at −1.0 V until 563 C (1.04 F mol^{-1}) had been consumed. Work-up afforded 3,6-dinitro-4,5-diphenyloctane (**18**, 0.84 g, 2.36 mmol, 84%) as colourless, oily and partially crystalline mixture of diastereomers. Flash-chromatographie with petroleum ether/acetone (10:1) and HPLC [column 1: petroleum ether/acetone (10:1)] allow to identify five diastereomers in a ratio of of **18a** : **b** : **c** : **d** : **e** ≈ 11:17:4:1:3 (^{1}H NMR).

Mp. (**18a**): 116-120 °C.- IR (KBr): ν (cm^{-1}) = 3050, 3010 (=CH), 2950, 2900, 2850 (CH), 1535 (NO_2), 1490, 1445, 1360 (NO_2), 1330, 1305, 1250, 1070, 910, 800, 770, 690 (=CH).

18a ^{1}H NMR ($CDCl_3$): δ (ppm) = 0.77 (t, $J_{1,2}$ = $J_{7,8}$ = 7.4 Hz, 6 H, 2 CH_3), 1.43 (ddq, $J_{1,2}$ = $J_{7,8}$ = 7.4 Hz, $J_{2A,2B}$ = $J_{7A,7B}$ = 14.8 Hz, $J_{2A,3}$ = $J_{6,7A}$ = 3.2 Hz, 2-, 7-CH_AH_B), 1.59 (ddq, $J_{1,2}$ = $J_{7,8}$ = 7.4 Hz, $J_{2A,2B}$ = $J_{7A,7B}$ = 14.8 Hz, $J_{2B,3}$ = $J_{6,7B}$ = 10.7 Hz, 2 H, 2-, 7-CH_AH_B), 3.42 (d, $J_{3,4}$ = $J_{5,6}$ = 10.9 Hz, 2 H, 4-, 5-H), 4.66 (ddd, $J_{2A,3}$ = $J_{6,7A}$ = 3.2 Hz, $J_{2B,3}$ = $J_{6,7B}$ = 10.7 Hz, $J_{3,4}$ = $J_{5,6}$ = 10.9 Hz, 2 H, 3-, 6-H), 7.1-7.5 (m, 10 H, aromat. H).

18b: δ (ppm) = 0.81 (t, $J_{1,2}$ = 7.4 Hz, 3 H, diastereotopic 1-CH_3), 1.07 (t, $J_{7,8}$ = 7.4 Hz, 3 H, diastereotopic 8-CH_3), 1.47 (ddq, $J_{1,2}$ = 7.4 Hz, $J_{2A,2B}$ = 14.8 Hz, $J_{2A,3}$ = 3.4 Hz, 1 H, diastereotopic 2-CH_AH_B), 1.58 (ddq, $J_{1,2}$ = 7.4 Hz, $J_{2A,2B}$, = 14.8 Hz, $J_{2B,3}$ = 10.1 Hz, 1 H, diastereotopic 2-CH_AH_B), 2.10 (ddq, $J_{7,8}$ = 7.4 Hz, $J_{7A,7B}$ = 14.7 Hz, $J_{6,7B}$ = 10.4 Hz, 1 H, diastereotopic 7-CH_AH_B), 2.47 (ddq, $J_{7,8}$ = 7.4 Hz, $J_{7A,7B}$ = 14.7 Hz, $J_{6,7A}$ = 3.1 Hz, 1 H, diastereotopic 7-CH_AH_B), 3.35 (dd, $J_{4,5}$ = 2.46 Hz, $J_{5,6}$ = 11.4 Hz, 1 H, diastereotopic 5-H), 3.74 (dd, $J_{3,4}$ = 11.4 Hz, $J_{4,5}$ = 2.46 Hz, 1 H, diastereotopic 4-H), 4.63 (ddd, $J_{5,6}$ = 11.4 Hz, $J_{6,7A}$ = 3.1 Hz, $J_{6,7B}$ = 10.4 Hz, 1 H, diastereotopic 6-H), 4.71 (ddd, $J_{2A,3}$ = 3.4 Hz, $J_{2B,3}$ = 10.1 Hz, $J_{3,4}$ = 11.4 Hz, 1 H, diastereotopic 3-H), 6.6-6.9, 7.1-7.4 (m, 10 H, aromat. H).

The assignment of the signals to distinct protons was achieved using decoupling experiments.

18c: δ (ppm) = 1.09 (t, $J_{1,2}$ = $J_{7,8}$ = 7.4 Hz, 6 H, 2 CH_3), 2.04 (ddq, $J_{1,2}$ = $J_{7,8}$ = 7.4 Hz, J_{2A2B} = J_{7A7B} = 14.5 Hz, $J_{2B,3}$ = $J_{6,7B}$ = 9.9 Hz, 3 H, 2-, 7- CH_AH_B), 2.26 (ddq, $J_{1,2}$ = $J_{7,8}$ = 7.4 Hz, $J_{2A,2B}$ = $J_{7A,7B}$ = 14.5 Hz, $J_{2A,3}$ = $J_{6,7A}$ = 3.6 Hz, 2 H, 2-, 7-CH_A,CH_B), 3.67 (dd, $J_{3,4}$ = $J_{5,6}$ = 8.2 Hz, $J_{4,5}$ = 1.78 Hz, 2 H, 4-, 5-H), 4.92 (ddd, $J_{3,4}$ = $J_{5,6}$ = 8.2 Hz, $J_{2A,3}$ = $J_{6,7A}$ = 3.6 Hz, $J_{2B,3}$ = $J_{6,7B}$ = 9.9 Hz, 2 H, 4-, 5-H), 6.65-6.80 (br m, 4 H, aromat. H), 7.10-7.25 (m, 6 H, aromat. H).

The signals were assigned by decoupling experiments.

18d: δ (ppm) = 0.65 (t, J = 7.25 Hz, 6 H, 2 CH_3), 1.7-1.9 (m, 4 H, 2 CH_2), 3.90-3.95 (m, 2 H, 4-, 5-H), 4.3-4.4 (m, 2 H, 3-, 6-H), 7.3-75 (m, 10 H, aromat. H).

18e: δ (ppm) = 0.61 (t, $J_{1,2}$ = 7.23 Hz, 3 H, diastereotopic 1-CH_3), 0.83 (t, $J_{7,8}$ = 7.35 Hz, 3 H, diastereotopic 8-CH_3), 1.45-1.90 (4 incompletely resolved ddq, J_{gem} = 14.5 and 14.6 Hz, 4 H,

2-, 7-CH_AH_B), 3.46 (dd, $J_{4,5}$ =10.9 Hz, $J_{5,6}$ = 4.1 Hz, 1 H, diastereotopic 5-H), 4.22 (dd, $J_{3,4}$ = 5.2 Hz, $J_{4,5}$ = 10.9 Hz, 1 H, diastereotopic 4-H), 4.28 (ddd, $J_{2A,3}$ = 2.1 Hz, $J_{2B,3}$ = 11.1 Hz, $J_{3,4}$ = 5.2 Hz, 1 H, diastereotopic 3-H), 4.41 (ddd, $J_{5,6}$ = 4.1 Hz, $J_{7A,3}$ = 5.2 Hz, $J_{7B,3}$ = 9.2 Hz, 1 H, diastereotopic 6-H), 7.1-7.3 (m, 4 H, aromat. H), 7.3-7.5 (m, 6 H, aromat. H).
The numbering is done according to this of **2e.**

^{13}C NMR ($CDCl_3$)
18a: δ (ppm) = 9.91 (q, C-1/-8), 25.86 (t, C-2/-7), 50.03 (d, C-4/-5), 91.49 (d, C- 3/-6), 128.18 (d, aromat. C-2 - C-6), 133.16 (s, aromat. C-1).
18b: δ (ppm) = 9.80, 9.95 (2 q, C-1/-8), 25.78, 26.00 (2 t, C-2/-7), 48.73, 49.71 (2 d, C-4/-5), 91.07, 91.38 (2 d, C-3/-6), 127.98, 128.11, 128.33 (3 d, aromat. C-2/-3/-5/-6), 130.01 (d, aromat. C-4), 133.26, 133.69 (2 s, aromat. C-1).
18c: δ (ppm) = 10.31 (q, C-1/-8), 25.69 (t, C-2/-7), 49.07 (d, C-4/-5), 90.54 (d, C-3/-6), 128.12, 128.23 (2 d, aromat. C-2/-3/-5/-6), 129.41 (d, aromat. C-4), 134.12 (s, aromat. C-1).
18d: δ (ppm) = 10.61 (q, C-1/-8), 21.12 (t, C-2/-7), 51.27 (d, C-4/-5), 88.92 (d, C- 3/-6), 128.54, 128.63, 128.86, 129.03 (d), 134.40 (s).
18e: δ (ppm) = 10.44, 10.81 (2 q, C-1/-8), 20.98, 24.88 (2 t, C-2/-7), 50.31, 51.31 (2 d, C-4/-5), 89.76, 91.70 (2 d, C-3/-6), 128.52, 128.84, 128.96, 129.09 (d), 134.03, 135.50 (2 s, aromat. C-1).
MS (GC/MS-coupling, EI, 70 eV):
m/z (%) = 356 (0.5) [M^+], 263 (1.2) [M^+-HNO_2-NO_2) 222 (3.8) [M^+- HNO_2-H_3CCH=$CHNO_2$, McLafferty], 221 (2.3) [M^+-HNO_2-$H_5C_2CHNO_2$, benzyl-cleav.], 178 (5.7) [M^+/2], 131 (12), 132 (100) [$C_6H_5C_4H_7^+$], 117 (40), 115 (15), 105 (10), 91 (28) [$C_7H_7^+$], 77 (5), 65 (4), 51 (3).
$C_{20}H_{24}N_2O_4$ (356.4) calc.: C 67.40, H 6.79, N 7.86; found: C 67.43, H 6.83, N 7.90.

3.7 Cathodic hydrodimerization of 9 to 19
According the GWP (chapt. 3.1) (*E*)-1-(4-methoxyphenyl)-2-nitroprop-1-ene (**9**, 0.95 g, 4.92 mmol) was electrolyzed at −1.1 V until 489 C (1.03 F mol^{-1}) were consumed. After work-up 3,4-bis-(4-methoxyphenyl)-2,5-dinitrohexane (**19**, 0.72 g, 1.85 mmol, 75%) were obtained as colourless, oily and partially crystallized mixture of diastereomers. Flash chromatography with petroleum ether/acetone (6:1) allowed to identify five diastereomers in a ratio of **19a** :**b**:**c**:**d**:**e**= 12:20:6:1:6 (^{1}H NMR).
IR (KBr): ν (cm^{-1}) = 3010 (=CH), 2930 (CH), 2840 (OCH_3), 1610 (C=C), 1540 (NO_2), 1505, 1445, 1355 (NO_2), 1240 (C-O-C), 1175, 1020, 825 (=CH, 1,4-disubstitution).

^{1}**H NMR** ($CDCl_3$):

19a: δ (ppm) = 1.23 (d, $J_{1,2}$= $J_{5,6}$ = 6.6 Hz, 6 H, 2 CH_3), 3.35 (d, $J_{2,3}$ = $J_{4,5}$ = 10.8 Hz, 2 H, 3-,4-H), 3.81 (s, 6 H, 2 OCH_3), 4.78 (dq, $J_{1,2}$ = $J_{5,6}$ = 6.6 Hz, $J_{2,3}$ = $J_{4,5}$ = 10.8 Hz, 2 H, 2-, 5-H), 6.6 - 6.9 (br d, 8 H, aromat. H).

19b: δ (ppm) = 1.26 (d, $J_{1,2}$ = 6.6 Hz, 3 H, diastereotopic 1-CH_3), 1.86 (d, $J_{5,6}$ = 6.5 Hz, 3 H, diastereotopic 6-CH_3), 3.26, 3.62 (2 dd, $J_{2,3}$ = $J_{4,5}$ = 11.18 Hz, $J_{3,4}$ = 2.65 Hz, 2 H, diastereotopic 3-, 4-H), 3.77, 3.81 (2 s, 6 H, 2 diastereotopic OCH_3), 4.72 (dq, $J_{4,5}$ = 11.18 Hz, $J_{5,6}$ = 6.5 Hz, 1 H, diastereotopic 5-H), 4.83 (dq, $J_{1,2}$ = 6.6 Hz, $J_{2,3}$ =11.18 Hz, 1 H, diastereotopic 2-H), 6.5 - 7.0, 7.1 - 7.4 (m, 8 H, aromat. H).

19c: δ (ppm) = 1.74 (d, $J_{1,2}$ = $J_{5,6}$ = 6.49 Hz, 6 H, 2 CH_3), 3.53 (dd, $J_{2,3}$ = $J_{4,5}$ = 5.95 Hz, $J_{3,4}$ = 1.8 Hz, 2 H, 3-, 4-H), 3.74 (s, 6 H, 2 OCH_3), 5.03-5.12 (m, 2 H, 2-, 5- H), 6.5-7.0 (m, 8 H, aromat. H).

19d: δ (ppm) = 1.27 (d, $J_{1,2}$ = $J_{5,6}$ = 6.74 Hz, 6 H, 2 CH_3), 3.82 (s, 6 H, 2 OCH_3), 4.00-4.05 (m, 2 H, 3-, 4-H), 4.45-4.55 (m, 2 H, 2-, 5-H), 7.1-7.4 (m, 8 H, aromat. H).

19e: δ (ppm) = 1.23 (d, $J_{1,2}$ = 6.64 Hz, 3 H, diastereotopic 1-CH_3), 1.28 (d, $J_{5,6}$ = 6.78 Hz, 3 H, diastereotopic 6-CH_3), 3.21 (dd, $J_{3,4}$ = 12.20 Hz, $J_{4,5}$ = 2.85 Hz, 1 H, diastereotopic 4-H), 3.81, 3.82 (2 s, 6 H, 2 diastereotopic OCH_3), 4.42 (dq, $J_{1,2}$ = 6.64 Hz, $J_{2,3}$ = 4.25 Hz, 1 H, diastereotopic 2-H), 4.48 (dd, $J_{2,3}$ = 4.25 Hz, $J_{3,4}$ = 12.20 Hz, 1 H, diastereotopic 3-H), 4.61 (dq, $J_{4,5}$ = 2.85 Hz, $J_{5,6}$ = 6.78 Hz, 1 H, diastereotopic 5-H), 6.9-7.0 (m, 4 H, aromat. H), 7.15-7.25 (m, 4 H, aromat. H).

The coupling constants are assigned following **2c**.

^{13}**C NMR** ($CDCl_3$):

19a: δ (ppm) = 18.80 (q, C-1/-6), 50.05 (d, C-3/-4), 55.16 (q, OCH_3), 85.08 (d, C- 2/-5), 113.49 (d, aromat. C-3/-5), 124.79 (s, aromat. C-1), 131.75 (broad d, aromat. C-2/-6), 159.30 (s, aromat. C-4).

19b: δ (ppm) = 19.00, 19.08 (2 q, C-1/-6), 48.81, 50.27 (2 d, C-3/-4), 55.09, 55.19 (2 q, OCH_3), 84.91, 85.18 (2 d, C-2/-5), 113.29, 113.74 (2 d, aromat. C-3/- 5), 124.90, 125.53 (2 s, aromat. C-1), 131.13 (broad d, aromat. C-2/-6), 159.29, 159.43 (2 s, aromat. C-4).

19c and **19d**: δ (ppm) = 11.43, 18.48 (2 q, C-1/-6), 49.06, 49.85 (2 d, C-3/-4), 55.0 (q, OCH_3), 83.72, 83.96 (2 d, C-2/-5), 113.52, 114.66 (2 d, aromat. C-3/-5), 124.50, 125.92 (2 s, aromat. C-1), 129.89, 130.33 (2 d, aromat. C-2/-6), 159 (s, aromat. C-4).

19e: δ (ppm) = 11.79, 17.37 (2 q, C-1/-6), 49.22, 51.49 (2 d, C-3/-4), 55.18, 55.19 (2 q, OCH_3), 82.61, 84.04 (2 d, C-2/-5), 114.38, 114.68 (2 d, aromat. C-3/- 5), 125.46, 126.28 (2 s, aromat. C-1), 130.33 (broad, aromat. C-2/-6), 159.58, 159.84 (2 s, aromat. C-4).

MS (GC/MS-coupling, EI, 70 eV):
m/z (%) = 388 (0.2) [M^+], 295 (0.5) [M^+-HNO_2-NO_2], 268 (3) [M^+- HNO_2-H_2C=$CHNO_2$, McLafferty], 207 (1.7) [M^+-HNO_2-H_3CCHNO_2, benzyl-cleav.], 194 (11) [$M^+/2$], 149 (12), 148 (100) [$MeOC_6H_4C_3H_5^+$], 147 (11), 121 (8) [$MeOC_7H_6^+$], 91 (5), 77 (6).
$C_{20}H_{24}N_2O_6$ (388.4) calc. C 61.85, H 6.23, N 7.21; found: C 61.85, H 6.27, N 7.12,

3.8 Cathodic hydrodimerization of 10 to 20

Using the GWP (chapt. 3.1) (*E*)-2-nitro-1-(4-trifluoromethylphenyl)-prop-1-ene (**10**, 1.00 g, 4.33 mmol) was electrolyzed at −0.85 V until 468 C (1.12 F mol^{-1}) were consumed to afford after work-up 3,4-bis(4-trifluoromethylphenyl)-2,5-dinitrohexane (**20**, 0.68 g, 1.46 mmol, 68%) as colourless crystalline mixture of diastereomers. Flash chromatography with petroleum ether/acetone (8:1) provides in a poor separation three fractions, whose further separation by HPLC [column 1, petroleum ether/diethyl ether (8:1)] allows the identification of five diastereomers in a ratio of about **20a**:**b**:**c**:**d**:**e** = 7:14:3:1:2 (^{1}H NMR).

IR (KBr): ν (cm^{-1}) = 3050 (=CH), 2960, 2820 (CH), 1625 (C=C), 1550 (NO_2), 1425, 1395, 1360 (NO_2), 1325 (C-F), 1160, 1110, 1070, 1020, 850 (=CH, 1,4-disubst.).

^{1}H NMR ($CDCl_3$):
20a: δ (ppm) = 1.26 (d, $J_{1,2}$ = $J_{5,6}$ = 6 6 Hz, 6 H, 2 CH_3), 3.57 (d, $J_{2,3}$ = $J_{4,5}$ = 10.8 Hz, 2 H, 3-, 4-H), 4.83(dq, $J_{1,2}$ = $J_{5,6}$ = 6.6 Hz, $J_{2,3}$ = $J_{4,5}$ = 10.8 Hz, 2 H, 2-, 5-H), 6.7-7.0, 7.4-7.7 (m, 8 H, aromat. H).
20b: δ (ppm) = 1.30 (d, $J_{1,2}$ = 6.63 Hz, 3 H, diastereotopic 1-CH_3), 1.93 (d, $J_{5,6}$ = 6.52 Hz, 3 H, diastereotopic 6-CH_3), 3.48, 3.84 (2 dd, $J_{2,3}$ = $J_{4,5}$ = 11.08 Hz, $J_{3,4}$ = 2.85 Hz, 2 H, diastereotopic 3-, 4-H), 4.76 (dq, $J_{4,5}$ = 11.08 Hz, $J_{5,6}$ = 6.52 Hz, 1 H, diastereotopic 5-H), 4.90 (dq, $J_{1,2}$ = 3.63 Hz, $J_{2,3}$ = 11.08 Hz, 1 H, diastereotopic 2-H), 6.6-6.9, 7.4-7.8 (m, 8 H, aromat. H).
20c: δ (ppm) = 1.63 (d, $J_{1,2}$ = $J_{5,6}$ = 6.51 Hz, 6 H, 2 CH_3) 3.74-3.78 (m, 2 H, 3-, 4-H), 5.4-5.5 (m, 2 H, 2-, 5-H), 6.9-7.0, 7.4-7.5 (2 br d, 8 H, aromat. H).

20d: δ (ppm) = 1.30 (d, $J_{1,2}$ = $J_{5,6}$ = 7.1 Hz, 6 H, 2 CH_3), 4.20-4.27 (m, 2 H, 3-, 4-H), 4.42-4.55 (m, 2 H, 2-, 5-H), 7.4-7.5, 7.7-7.8 (2 br d, 8 H, aromat. H).

20e: δ (ppm) = 1.26 (d, $J_{1,2}$ = 6.8 Hz, 3 H, diastereotopic 1-CH_3), 1.32 (d, $J_{5,6}$ = 6.8 Hz, 3 H, diastereotopic 6-CH_3), 3.40 (dd, $J_{3,4}$ = 12.17 Hz, $J_{4,5}$ = 3.06 Hz, 1 H, diastereotopic 4-H), 4.40 (dq, $J_{1,2}$ = 6.8 Hz, $J_{2,3}$ = 4.54 Hz, 1 H, diastereotopic 2-H), 4.56 (dq, $J_{4,5}$ = 3.06 Hz, $J_{5,6}$ = 6.8 Hz, 1 H, diastereotopic 5-H), 4.66 (dd, $J_{2,3}$ = 4.54 Hz, $J_{3,4}$ = 12.17 Hz, 1 H, diastereotopic 3-H), 7.4-7.8 (m, 8 H, aromat. H). The numbering follows this of **2e**.

^{13}C NMR ($CDCl_3$):

20a: δ (ppm) = 18.70 (q, C-1/-6), 50.57 (d, C-3/-4), 84.34 (d, C-2/-5), 125.44 (CF_3), 129.48 (d), 138.11 (s).

20b: δ (ppm) = 18.85, 18.94 (2 q, C-1/-6), 49.32, 50.68 (2 d, C-3/-4), 84.08, 84.43 (2 d, C-2/-5), 125.23, 125.67 (CF_3), 130.36 (d), 136.91, 137.49 (s).

20c: δ (ppm) = 17.78 (q, C-1/-6), 50.69 (d, C-3/-4), 82.24 (d, C-2/-5), 126.46 (CF_3), 131.06 (d), 136.76 (s).

The resonances of **20d** and **20e** cannot be unequivocally assigned due to their low intensity in the mixture of diastereomers. All recognizable resonance signals, however, are found in the expected areas (11-20, 49-52, 82-85 and 121-140 ppm).

MS (direct inlet, EI, 70 eV):

m/z (%) = 464 (5) [M^+], 445 (8) [M^+-F], 398 (8) [M^+-HNO_2-F], 371 (6) [M^+-HNO_2-NO_2], 344 (8) [M^+-HNO_2-H_2C=$CHNO_2$, McLafferty], 343 (8) [M^+-HNO_2-H_3CCHNO_2, benzyl-cleav.], 232 (7) [M^+/2], 187 (25), 186 (100) [$F_3CC_6H_4C_3H_5^+$], 173 (47), 159 (33) [$F_3CC_7H_6^+$], 117 (62), 115 (33).

$C_{20}H_{18}F_6N_2O_4$ (464.36) calc. C 51.89, H 3.98, N 6.01, found C 51.73, H 3.91, N 6.03. A correct fluorine value was not obtained in spite of multiple purifications by HPLC.

HRMS (direct inlet, EI, 70 eV):

$C_{20}H_{18}F_6N_2O_4$: calc. 464.11707 found: 464.11647.

3.9 Cathodic hydrodimerization of 11 to 21

Using the GWP (chapt. 3.1) (*E*)-1-(furan-2-yl)-2-nitroprop-1-ene (**11**, 0.77 g, 5.03 mmol) were electrolyzed at −1.1 V to afford after a current consumption of 502 C (1.03 F mol^{-1}) 3,4-bis(furan-2-yl)-2,5-dinitrohexane (**21**, 0.66 g, 2.14 mmol, 85%) as colourless, oily and partially crystalline mixture of diastereomers. By HPLC [column 2, petroleum ether/acetone (8 : 1)] five diastereomers in a ratio of **21a**:**b**:**c**:**d**:**e** ≈ 11:16:3:1:2 (^{1}H NMR) can be identified.

Mp.: **21a:** 75-78 °C

IR (KBr): ν (cm^{-1}) = 3030 (=CH), 2930, 2910 (CH), 1620 (C=C), 1545 (NO_2), 1510, 1455, 1385, 1360 (NO_2), 1220, 1195, 1145, 1010, 930, 870, 745 (=CH).

^{1}H NMR ($CDCl_3$):

21a: δ(ppm) = 1.30 (d, $J_{1,2}$ = $J_{5,6}$ = 6.63 Hz, 6 H, 2 CH_3), 3.51 (d, $J_{2,3}$ = $J_{4,5}$ = 10.54 Hz, 2 H, 3-, 4-H), 4.86 (dq, $J_{1,2}$ = $J_{5,6}$ = 6.63 Hz, $J_{2,3}$ = $J_{4,5}$ =10.54 Hz, 2 H, 2-, 5-H), 6.34 (dd, J = 3.26 Hz, ^4J = 0.86 Hz, 2 H, O-CH=CH-C*H*), 6.38 (dd, J = 1.87, 3.26 Hz, 2 H, O-CH=C*H*-CH), 7.31 (dd, J = 1.87 Hz, ^4J = 0.86 Hz, 2 H, O-C*H*=CH-CH).

21b: δ = 1.32, 1.75 (2 d, $J_{1,2}$ = $J_{5,6}$ = 6.62 Hz, 6 H, diastereotopic 1-/6- CH_3), 3.56, 3.81 (2 dd, $J_{2,3}$ = $J_{4,5}$ = 10.78 Hz, $J_{3,4}$ = 3.80 Hz, 2 H, diastereotopic 3-, 4-H), 4.74, 4.82 (2 dq, $J_{1,2}$ = $J_{5,6}$ = 6.62 Hz, $J_{2,3}$ = $J_{4,5}$ = 10.78 Hz, 2 H, diastereotopic 2-, 5-H), 6.07, 6.16 (2 d, J = 3.3 Hz, 2 H, diastereotopic O-CH=CH-C*H*), 6.31, 6.35 (2 dd, J = 1.85, 3.3 Hz, 2 H, diastereotopic O-CH=C*H*-CH), 7.32, 7.33 (2 m, 2 H, diastereotopic O-C*H*=CH-CH).

21c: δ (ppm) = 1.41 (d, $J_{1,2}$ = $J_{5,6}$ = 6.51 Hz, 6 H, 2 CH_3), 3.78-3.85 (not completely resolved dd, 2 H, 3-, 4-H), 4.8-4.9 (not completely resolved dq, 2 H, 2-, 5- H), 5.97-6.0, 6.23-6.28, 7.28-7.31 (3 m, 6 H, O-CH=CH-CH).

The multiplicities are assumed in analogy to these of **2c - 20c**. An exact assignment is due to the signal overlap with **21b** not possible.

21d: δ (ppm) = 1.41 (d, $J_{1,2}$ = $J_{5,6}$ = 6.57 Hz, 6 H, 2 CH_3), 4.25-4.37 (m, 4 H, 2- /3-/4-/5-H), 6.28-6.35, 6.35-6.41, 7.45-7.52 (3 m, 6 H, O-CH=CH-CH).

21e: δ (ppm) = 1.36, 1.41 (2 d, J = 6.7, 6.8 Hz, 6 H, diastereotopic 1-/6-CH_3), 3.67 (dd, $J_{3,4}$ = 12.04 Hz, $J_{4,5}$ = 3.29 Hz, 1 H, diastereotopic 4-H), 4.32 (dq, $J_{1,2}$ = 6.74 Hz, $J_{2,3}$ = 4.45 Hz, 1 H, diastereotopic 2-H), 4.43 (dq, $J_{4,5}$ = 3.29 Hz, $J_{5,6}$ = 6.80 Hz, 1 H, diastereotopic 5-H), 4.61 (dd, $J_{2,3}$ = 4.45 Hz, $J_{3,4}$ = 12.04 Hz, 1 H, diastereotopic 3-H), 6.33-6.42 (m, 4 H, O-CH=CH-C*H*), 7.41-7.46 (m, 2 H, O-C*H*=CH-CH).

^{13}C NMR ($CDCl_3$):

21a: δ (ppm) = 18.81 (q, C-1/-6), 43.86 (d, C-3/-4), 83.28 (d, C-2/-5), 110.54, 111.55 (2 d, furyl-C-3/-4), 142.77 (d, furyl-C-5), 147.03 (s, furyl-C-2).

21b: δ (ppm) = 18.33, 18.83 (2 q, C-1/-6), 43.21, 43.61 (2 d, C-3/-4), 83.58, 83.82 (2 d, C-2/-5), 110.03, 110.08, 110.48, 110.66 (4 d, furyl-C-3/-4), 142.59, 142.67 (2 d, furyl-C-5), 147.54, 148.28 (2 s, furyl-C-2).

21c: δ (ppm) = 18.19 (q, C-1/-6), 43.84 (d, C-3/-4), 83.52 (d, C-2/-5), 109.27, 110.40 (2 d, furyl-C-3/-4), 142.48 (d, furyl-5-C), 148.6 (s, furyl-C-2).

The resonances of **21d** cannot be unequivocally assigned due to their low intensity in the mixture of diastereomers. All recognizable resonance signals, however, are found in the expected areas (13-19, 43-45, 82-84, 109-112 and 142-149 ppm).

21e: δ (ppm) = 13.57, 17.10 (2 q, C-1/-6), 43.58, 44.64 (2 d, C-3/-4), 82.27, 83.91 (2 d, C-2/-5), 110.58, 110.82, 110.98, 111.83 (4 d, furyl-C-3/-4), 143.11, 143.48 (2 d, furyl-C-5), 147.9, 148.5 (2 s, furyl-C-2).

MS (GC/MS-coupling, EI, 70 eV):

m/z (%) = 308 (0) [M^+], 262 (3.7) [M^+-NO_2], 215 (0.6) [M^+-HNO_2-NO_2], 188 (1) [M^+-HNO_2-H_2C=$CHNO_2$, McLafferty], 187 (3) [M^+-HNO_2-H_3CCHNO_2, benzyl-cleavage], 154 (6) [M^+/2], 108 (100) [$C_4H_3OC_3H_5^+$], 79 (14).

$C_{14}H_{16}N_2O_6$ (308.3) Calc. 54.54, H 5.23, N 9.09; found: C 54.54, H 5.31, N 9.11.

3.10 Cathodic hydrodimerization of 12 to 22

Using the GWP (chapt. 3.1) (*E*)-2-nitro-1-(pyrrol-2-yl)prop-1-ene (**12**, 0.89 g, 5.85 mmol) was electrolyzed at −1.15 V to afford after consumption of 589 C (1.04 F·mol^{-1}) 2,5-dinitro-3,4-bis(pyrrol-2-yl)hexane (**22**, 0.65 g, 2.12 mmol, 73%) as yellowish, oily and partially crystalline mixture of diastereomers. The product discolours in the air to brown. Different from the GWP the electrolysis was performed in a divided cell (Figure S1). In the undivided cell the electrolyte discolours to black, presumably by oxidation of the pyrrole group, and the yield decreases to 60 % of **22**.

Flash chromatography with petroleum ether/dichloromethane/diethyl ether(5:4:2, v/v/v) and HPLC [column 2, petroleum ether/acetone (4:1)] allows to identify five diastereomers in a ratio of about **22a** : **b** : **c** : **d** : **e** ≈ 1.7:3.2:1.7:1:5.5 (^{1}H NMR).

IR (KBr):

ν(cm^{-1}) = 3550, 3400 (NH), 3100 (=CH), 2930 (CH), 1610 (C=C), 1540 (NO_2), 1445, 1385, 1355 (NO_2), 1275, 1085, 1025, 960, 860, 720 (=CH).

^{1}H NMR ($CDCl_3$):

22a: δ (ppm) = 1.28 (d, $J_{1,2} = J_{5,6}$ = 6.6 Hz, 6 H, 2 CH_3), 3.35 (d, $J_{2,3} = J_{4,5}$ = 10.6 Hz, 2 H, 3-, 4-H), 4.72 (dq, $J_{1,2} = J_{5,6}$ = 6.6 Hz, $J_{2,3} = J_{4,5}$ =10.6 Hz, 2 H, 2-, 5-H), 5.82-5.89 (m, 2 H, N-CH=CH-C*H*), 6.16-6.22 (m, 2 H, N-CH=C*H*-CH), 6.76-6.80 (m, 2 H, N-C*H*=CH-CH), 8.1-8.3 (br s, 2 H, NH).

22b: δ (ppm) = 1.18 (d, $J_{1,2}$ = 6.57 Hz, 3 H, diastereotopic 1-CH_3), 1.70 (d, $J_{5,6}$ = 6.61 Hz, 3 H, diastereotopic 6-CH_3), 3.38 (dd, $J_{3,4}$ = 3.35 Hz, $J_{4,5}$ = 10.95 Hz, 1 H, diastereotopic 4-H), 3.57

(dd, $J_{2,3}$ = 11.12 Hz, $J_{3,4}$ = 3.35 Hz, 1 H, diastereotopic 3-H), 4.65 (dq, $J_{4,5}$ = 10.95 Hz, $J_{5,6}$ = 6.61 Hz, 1 H, diastereotopic 5-H), 4.78 (dq, $J_{1,2}$ = 6.57 Hz, $J_{2,3}$ = 11.12 Hz, 1 H, diastereotopic 2-H), 5.95-6.0, 6.05-6.1 (2 m, 2 H, diastereotopic N-CH=CH-C*H*), 6.1-6.17, 6.17-6.21 (2 m, 2 H, diastereotopic N-CH=C*H*-H), 6.68—6.72, 6.77-6.82 (2 m, 2 H, diastereotopic N-C*H*=CH-CH), 7.7-7.9, 8.5-87 (2 br s, 2 H, diastereotopic NH).

22c: δ (ppm) = 1.74 (d, $J_{1,2}$ = $J_{5,6}$ = 6.54 Hz, 6 H, 2 CH_3), 3.55-3.64 (m, 2 H, 3-, 4-H), 4.78-4.9 (m, 2 H, 2-, 5-H), 5.92-6.0, 6.1-6.16, 6.63-6.7 (3 m, 6 H, N-CH=CH-CH), 7.8-8.0 (br s, NH).

22d (D_3CCOCD_3): δ (ppm) = 1.44 (d, $J_{1,2}$ = $J_{5,6}$ = 6.78 Hz, 6 H, 2 CH_3), 4.18- 4.22 (m, 2 H, 3-/4-H), 4.45-4.55 (m, 2 H, 2-/5-H), 6.05-6.2 (m, 4 H, N-CH=C*H*-C*H*), 6.75-6.85 (m, 2 H, N-CH=C*H*-CH), 10.1-10.3 (br s, 2H, NH).

22e (D_3CCOCD_3): δ (ppm) = 1.29 (d, $J_{5,6}$ = 6.75 Hz, 3 H, diastereotopic 6-CH_3), 1.32 (d, $J_{1,2}$ = 6.69 Hz, 3 H, diastereotopic 1-CH_3), 3.59 (dd, $J_{3,4}$ = 12.51 Hz, $J_{4,5}$ = 3.18 Hz, 1 H, diastereotopic 4-H), 4.35 (dq, $J_{1,2}$ = 6.69 Hz, $J_{2,3}$ = 3.95 Hz, 1 H, diastereotopic 2-H), 4.53(dd, $J_{2,3}$ = 3.95 Hz, $J_{3,4}$ = 12.51 Hz, 1 H, diastereotopic 3-H), 4.66 (dq, $J_{4,5}$ = 3.18 Hz, $J_{5,6}$ = 6.75 Hz, 1 H, diastereotopic 5-H), 6.0-6.1 (m, 4 H, N-C*H*=C*H*-CH), 6.7-6.8 (m, 2 H, N-C*H*=CH-CH), 9.8-10.0 (m, 2 H, NH)

^{13}C NMR ($CDCl_3$):

22a: δ (ppm) = 18.58 (q, C-1/-6), 44.78 (d, C-3/-4), 84.93 (d, C-2/-5), 108.88, 110.31 (2 d, pyrrole-C-3/-4), 118.52 (d, pyrrole-C-5), 123.14(s, pyrrole-C-2).

22b: δ (ppm) = 18.38, 18.85 (2 q, C-1/-6), 43.41, 44.62 (2 d, C-3/-4), 84.92, 84.99 (2 d, C-2/-5), 108.29, 108.37, 109.10, 111.24 (4 d, pyrrole-C-3/-4), 118.75, 119.54 (2 d, pyrrole-C-5), 122.53, 125.15 (2 s, pyrrole-C-2).

22c: δ (ppm) = 18.51 (q, C-1/-6), 44.34 (d, C-3/-4), 85.04 (d, C-2/-5), 108.77, 109.55 (2 d, pyrrole-C-3/-4), 118.83 (d, pyrrole-5-C), 123.99 (s, pyrrole-C-2).

22d: δ (ppm) = 12.75 (q, C-1/-6), 44.04 (d, C-3/-4), 83.32 (d, C-2/-5), 108.88, 109.35 (2 d, pyrrole-C-3/-4), 118.72 (d, pyrrole-5-C), 123.25 (s, pyrrole-C-2).

22e (D_3CCOCD_3): δ (ppm) = 12.07, 17.11 (2 q, C-1/-6), 45.14, 45.98 (2 d, C-3/- 4), 83.23, 83.82 (2 d, C-2/-5), 107.91, 108.23, 108.34 (3 d, pyrrole-C-3/-4), 118.08, 118.56 (2 d, pyrrole-C-5), 124.53, 125.20 (2 s, pyrrole-C-2).

MS (GC/MS-coupling, EI, 70 eV):

m/z (%) = 306 (0) [M^+], 259 (8.8) [M^+-HNO_2], 215 (1.2) [M^+-HNO_2-NO_2], 185 (3.4) [M^+-HNO_2-H_3CCHNO_2, benzyl-cleav.], 153 (6.3) [M^+/2], 108 (18), 107 (100) [$C_4H_4NC_3H_5^+$], 106 (23), 80 (21), 79 (10).

$C_{14}H_{18}N_4O_4$ (306.3) Calc. C 54.89, H 5.92, N 18.29; found: C 54.96, H 5.91, N 18.13.

3.11 Cathodic hydrodimerization of 13 to 23

Using the GWP (chapt. 3.1) (*E*)-1-(2-chlorophenyl)-2-nitroprop-1-ene (**13**, 1.02 g, 5.14 mmol) was electrolyzed at −0.95 V until 506 C (1.02 F mol^{-1}) were consumed affording 3,4-bis(2-chloropheny1)-2,5-dinitro-hexane (**23**, 0.85 g, 2.14 mmol, 83 %) as colorless partially crystalline mixture of diastereomers. Flash chromatography with petroleum ether/dichloromethane (1:1) followed by further separations of the fractions by HPLC allows to identify five diastereomers. The portion of **23a** and **23b** (1:1) amounts to about 80% of the total mixture. The residual 20% contain the other three diastereomers; their ratio **23c**:**d**:**e** cannot be determined exactly because of overlapping signals in the ^{1}H NMR.

IR (KBr):
ν (cm^{-1}) = 3050 (=CH), 2970, 2910, 2860 (CH), 1610 (C=C), 1525 (NO_2), 1470, 1430, 1385, 1350 (NO_2), 1330, 1310, 1280, 1250, 1220, 1180, 1110, 1085, 1030 (C-Cl), 855, 740 (=CH, 1,2-disubstitution), 680.
^{1}H NMR ($CDCl_3$):
23a: δ (ppm) = 1.56 (d, $J_{1,2}$ = $J_{5,6}$ = 6.61 Hz, 6 H, 2 CH_3), 4.50-4.58 (m, 2 H, 3-, 4-H), 5.6-5.7 (m, 2 H, 2-, 5-H), 6.85-6.93 (m, 2 H, aromat. 6-H), 6.97-7.08 (m, 4 H, aromat. 4-, 5-H), 7.13-7.21 (m, 2 H, aromat. 3-H).
23b: δ (ppm) = 1.32 (d, $J_{1,2}$ = 6.8 Hz, 3 H, diastereotopic 1-CH_3), 1.96 (d, $J_{5,6}$ = 6.5 Hz, 3 H, diastereotopic 6-CH_3), 4.34 (dd, $J_{3,4}$ = 3.41 Hz, $J_{4,5}$ = 9.6 Hz, 1 H, diastereotopic 4-H), 4.70 (dd, $J_{2,3}$ = 9.9 Hz, $J_{3,4}$ = 3.41 Hz, 1 H, diastereotopic 3-H), 4.98 (dq, $J_{4,5}$ = 9.6 Hz, $J_{5,6}$ = 6.5 Hz, 1 H, diastereotopic 5-H), 5.12 (dq, $J_{1,2}$ = 6.8 Hz, $J_{2,3}$ = 9.9 Hz, 1 H, diastereotopic 2-H); signals of aromatic ring: ring 1: 6.11 (dd, *J* = 7.9 Hz, ^4J = 1.5 Hz, 1 H, 6-H), 6.88 (ddd, *J* = 7.9 and 7.6 Hz, ^4J = 1.3 Hz, 1 H, 5-H), 7.15 (ddd, *J* = 8.0 and 7.6 Hz, ^4J = 1.5 Hz, 1 H, 4-H), 7.42 (dd, *J* = 8.0 Hz, ^4J = 1.3 Hz, 1 H, 3-H); ring 2: 6.26 (dd, *J* = 7.9 Hz, ^4J = 1.5 Hz, 1H, 6-H), 6.82 (ddd, *J* =7.9 and 7.6 Hz, ^4J = 1.3 Hz, 1 H, 5-H), 7.08 (ddd, *J* = 8.0 and. 7.6 Hz, ^4J = 1.5 Hz, 1 H, 4-H), 7.35 (dd, *J* = 8.0 Hz, ^4J = 1.3 Hz, 1 H, 3-H). The single signals were assigned with decoupling experiments.

23c: δ (ppm) = 1.37 (d, $J_{1,2} = J_{5,6}$ = 6.9 Hz, 6 H, 2 CH_3), 4.74 (dd, J = 4.9, 2.0 Hz, 2 H, 3-, 4-H), 4.95-5.05 (m, 2 H, 2-, 5-H), 6.75-6.80 (m, 2 H, aromat. 6-H), 6.95-7.10 (m, 4 H, aromat. 4-, 5-H), 7.22-7.30 (m, 2 H, aromat. 3-H).

The signals of the diastereomers **23d** and **23e** can not be assigned unequivocally due to their low intensity in the mixtures.

23d + **23e**: δ (ppm) = 1.19, 1.25, 1.36 (3 d,J = 6.7 and 6.8 Hz, Σ = 6 H, CH_3), 3.9-4.0, 4.3-4.6, 4.9-5.0, 5.6-5.7 (m, Σ = 4 H, 2-, 3-, 4-, 5-H), 6.8-7.5 (m, 8 H, aromat. H).

^{13}C NMR ($CDCl_3$):

23a: δ (ppm) = 16.62 (q, C-1/-6), 45.55 (d, C-3/-4), 82.37 (d, C-2/-5), 128.29, 128.86, 129.06, 129.31 (4 d, aromat. C-3/-4/-5/-6), 133.04, 135.01 (2 s, aromat. C-1/-2).

23b: δ (ppm) = 18.62, 19.02 (2 q, C-1/-6), 44.51, 46.74 (2 d, C-3/-4), 84.80, 85.02 (2 d, C-2/-5), 126.03, 126.19, 129.05, 129.20, 129.76, 129.97, 130.07, 131.33 (8 d, aromat. C-3/-4/-5/-6), 132.80, 133.14, 134.87, 135.10 (4 s, aromat. C-1/-2).

23c: δ (ppm) = 17.22 (q, C-1/-6), 46.37 (d, C-3/-4), 84.75 (d, C-2/-5), 128.37, 128.99, 129.85, 130.22 (4 d, aromat. C-3/-4/-5/-6), 133.53, 135.27 (2 s, aromat. C-1/-2).

The remaining diastereomers cannot be clearly assigned by ^{13}C NMR due to their low portion in the mixture of diastereomers. However, all visible signals are found in the expected areas (14-20, 44-47, 82-85 and 126-135 ppm).

MS (GC/MS-coupling, EI, 70 eV): m/z (%) = 400/398/396 (0.5/2.5/3.8) [M^+], 350 (0.2) [M^+-NO_2] 315 (1.3) [M^+-NO_2-Cl], 307/305/303 (0.4/2.0/3.1) [M^+-HNO_2-NO_2], 279/277/275 (0.6/2.5/4.1) [M^+-HNO_2-H_3CCHNO_2, benzyl-cleav.], 154/152 (38/100) [$ClC_6H_4C_3H_5^+$], 139 (32), 127/125 (9/22) [$ClC_7H_6^+$], 117 (30), 115 (22).

$C_{18}H_{18}Cl_2N_2O_4$ (397.26) Calc. C 54.42, H 4.57, Cl 17.85, N 7.05; found C 54.49, H 4.57, Cl 17.93, N 7.15.

3.12 Attempted cathodic hydrodimerization of 14 to 26

Using the GWP (chapt. 3.1) (*E*)-1-(2,6-dichlorophenyl)-2-nitroprop-1-ene (**14**, 1.18 g, 5.08 mmol) was electrolyzed at −1.1 V until 1013 C (2.07 F mol^{-1}) were consumed.

The current/time behaviour deviates significantly from the expectation. Only after consumption of more than 2 F/mol the current decreases to 15 mA. After a work-up with water no **26** could be detected in the diethyl ether extract. Flash chromatography with petroleum ether/diethyl ether (4:1) and petroleum ether/acetone (8:1) afforded pure (*E/Z*)-(2,6-dichlorophenyl)acetonoxime (**24**, 0.41 g, 1.90 mmol, 37%) as colorless crystals and 1-(2,6-dichlorophenyl)-2-nitropropan-1-ol (**25**, 0.39 g,1.56 mmol, 31%) as colorless oil.

Oxime **24**:

IR (KBr):

ν (cm^{-1}) = 3400-3150 (OH), 3050 (=CH), 2920 (CH), 1675 (C=N), 1580, 1560 (C=C), 1430, 1370, 1215, 1165, 1140, 1080 (C-Cl), 1010, 955, 885, 820, 760 (=CH, 1,2,3-trisubstitution).

^{1}H NMR ($CDCl_3$):

δ (ppm) = 1.61, 1.93 (2 s, Σ = 3 H, *Z*-, *E*-CH_3), 3.85, 4.12 (2 s, Σ = 2 H, *E*-, *Z*-CH_2), 7.1-7.4 (m, 3 H, aromat. H).

^{13}C NMR ($CDCl_3$):

E-**24**: δ (ppm) = 13.59 (q, CH_3), 37.12 (t, CH_2), 128.10, 128.45 (2 d, aromat. C-3/- 4/-5), 132.93 (s, aromat. C-1), 135.96 (s, aromat. C-2/-6), 154.34 (s, C=N).

The signals of *Z*-**24** are not recognizable due to their low intensity.

MS (GC/MS-coupling, EI, 70 eV):

m/z (%) = 221/219/217 (1/7/11) [M^+], 203/201/199 (1/3/5) [M^+-H_2O], 184/182 (44/100) [M^+-Cl], 163/161/159 (4/22/34) [$Cl_2C_7H_5^+$], 147 (17) [M^+-2 Cl], 140 (14), 125 (22), 123 (24), 89 (28), 63 (19), 58 (60) [CH_3-C=NOH$^+$, α-cleavage].

$C_9H_9Cl_2NO$ (218.1) Calc. C 49.57, H 4.16, CI 32.51, N 6.42; found: C 49.45, H 4.24, Cl 32.51, N 6.44.

1-(2,6-Dichlorophenyl)-2-nitropropan-1-ol (**25**)

The nitroalcohol **25** is a mixture of two diastereomers **25a** and **25b** in the ratio 7.5 : 1 (^{1}H-NMR).

IR (Film):

ν (cm^{-1}) = 3600-3200 (OH), 3080 (=CH), 2980, 2940, 2870 (CH), 1545 (NO_2), 1430, 1380, 1350 (NO_2), 1290, 1175, 1145, 1045 (C-Cl), 1015, 905, 860, 840, 765 (=CH, 1,2,3-trisubstitution), 730.

^{1}H NMR ($CDCl_3$):

25a: δ (ppm) = 1.37 (d, $J_{2,3}$ = 6.9 Hz, 3 H, CH_3), 3.13 (br d, $J_{1,OH}$ = 9 Hz, 1 H, OH, with D_2O exchangeable), 5.48 (dq, $J_{1,2}$ = 10.0 Hz, $J_{2,3}$ = 6.9 Hz, 1 H, 2- H), 6.00 (pseudo-t, after D_2O-exchange: d, $J_{1,2}$ = 10.0 Hz, 1 H, 1-H), 7.2-7.5 (m, 3 H, aromat. H).

25b: δ (ppm) = 1.81 (d, $J_{2,3}$ = 6.6 Hz, 3 H, CH_3), 3.11 (br d, $J_{1,OH}$ = 9.7 Hz, 1 H, OH, with D_2O exchangeable), 5.39 (dq, $J_{1,2}$ = 8.3 Hz, $J_{2,3}$ = 6.6 Hz, 1 H, 2-H), 5.80 (dd, after D_2O-exchange: d, $J_{1,2}$ = 8.3 Hz, 1 H, 1-H), 7.2-7.5 (m, 3 H, aromat. H).

The assignment of the signals to the structure was done with decoupling experiments.

^{13}C NMR ($CDCl_3$):

δ (ppm) = 15.93, 16.07 (2 q, **a**-, **b**-CH_3), 72.73 (d, C-1), 84.30, 85.83 (2 d, **b**-, **a**- C-2), 129.70 (br d), 130.36 (d), 130.56 (d), 132.45 (s), 134.9 (s).

MS (direct inlet, EI, 70 eV):

m/z (%) = 253/251/249 (3/12/16) [M^+], 207/205/203 (6/16/19) [M^+- NO_2], 206/204/202 (7/14/20) [M^+-HNO_2], 179/177/175 (20/80/100) [$Cl_2C_7H_4OH^+$, α-cleavage], 173 (86) [$Cl_2C_6H_3CO^+$], 149/147/145 (28/37/45) [$Cl_2C_6H_3^+$], 125 (59), 111 (76), 85 (46), 75 (93), 74 (70), 57 (42) [M^+- HNO_2.- $Cl_2C_6H_3$, α-cleavage].

HRMS (direct inlet, EI, 70 eV): $C_9H_9Cl_2NO_3$ Calc. 248.9960 Found 248.9954.

3.13 Cathodic hydrodimerization of 15 to 29

Using the GWP (chapt. 3.1) (*E*)-1-(2,6-difluorophenyl)-2-nitroprop-1-ene (**15**, 0.74 g, 3.72 mmol) were electrolyzed at −0.95 V until 483 C (1.35 F mol^{-1}) were consumed.

Flash chromatography with petroleum ether/acetone (6:1) afforded 3,4-bis(2,6-difluorophenyl)-2,5-dinitrohexane (**29**, 0.30 g, 0.75 mmol, 40%) as colourless, crystalline mixture of diastereomers, 1-(2,6-difluorophenyl)-2-nitropropan-1-ol (**28**, 0.15 g, 0.69 mmol, 19%) as colourless oil and (*E/Z*)-(2,6-difluorophenyl)acetonoxime (**27**, 0.04 g, 0.22 mmol, 6%).

3,4-Bis-(2,6-difluorophenyl-2,5-dinitrohexane (**29**)

IR (KBr):

ν (cm^{-1}) = 3080 (=CH), 2970, 2920, 2870 (CH), 1615, 1580 (C=C), 1540 (NO_2), 1460, 1380, 1350 (NO_2), 1215 (C-F), 970, 775 (=CH, 1,2,3-trisubstitution).

^{1}H NMR ($CDCl_3$):

δ (ppm) = 1.2-2.0 (m, 6 H, 2 CH_3), 3.7-5.6 (m, 4 H, 2-, 3-, 4-, 5-H), 6.5-7.5 (m, 6 H, aromat. H).

^{13}C NMR ($CDCl_3$):

Due to the overlap of the signal of several diastereomers only the regions of the signals are given. These regions are distinctive for the carbon frame of **29** (see chapters 3.4.-3.12.).

δ (ppm) = 16.1-19.7 (q, C-1/-6), 39.3-42.7 (d, C-3/-4), 81.1-85.2 (d, C-2/-5), 110.8-112.9 (d, aromat. C-3/-5), 128.4-131.5 (s and d, aromat. C-1/-4), 160- 164 (CF).

MS (GC/MS-Kopplung, EI, 70 eV):

m/z (%) = 400 (0.5) [M^+], 307 (5.5) [M^+-HNO_2-NO_2], 280 (1.5) [M^+- HNO_2-H_2C=$CHNO_2$, McLaffertyl], 279 (3.8) [M^+-HNO_2-H_3CCHNO_2, benzyl- cleavage], 200 (3) [M^+/2], 154 (100) [$F_2C_6H_3C_3H_5^+$], 141 (24), 127 (31) [$F_2C_7H_5^+$].

$C_{18}H_{16}F_2N_2O_4$ (400.3): Calc. C 54. 01, H 4.03, F 19.0, N 7.00; found: C 54.08, H 4.05 F 19.2 N 6.94.

(*E/Z*)-(2,6-Difluorophenyl)acetonoxime (**27**)

^{1}H NMR ($CDCl_3$):

δ (ppm) = 1.75, 1.90 (2 s, Σ = 3 H, *Z*-, *E*-CH_3), 3.57, 3.80 (2 s, Σ = 2 H, *E*-, *Z*-CH_2), 6.82-6.98 (m, 2 H, aromat.H), 7.15-7.30 (m, 1 H, aromat. H), 8.3-85 (br s, 1 H, OH).

MS (GC/MS-coupling, EI, 70 eV):

m/z (%) = 185 (10) [M^+], 168 (6) [M^+-OH], 167 (12) [M^+-H_2O], 166 (17) [M^+-F], 165 (76) [M^+-F-H], 152 (30), 128 (27), 127 (100) [$F_2C_7H_5^+$], 126 (21), 101 (17), 58 (81) [CH_3-C=NOH^+, α-cleavage].

HRMS (GC/MS-coupling, EI, 70 eV): $C_9H_9F_2NO$: Calc. 185.0652, Found: 185.0614.

1-(2,6-Difluorophenyl)-2-nitropropan-1-ol (**28**)

The nitroalcohol **28** is a mixture of two diastereomers **28a** and **28b** in the ratio 5.9 : 1 (^{1}H NMR).

IR (Film):

ν (cm^{-1}) = 3650-3200 (OH), 3100 (=CH), 2990, 2940, 2900 (CH), 1620, 1590 (C=C), 1545 (NO_2), 1465, 1385, 1350 (NO_2), 1270, 1220 (C-F), 1190, 1020, 980, 865, 780 (=CH, 1,2,3-trisubstitution), 725.

^{1}H NMR ($CDCl_3$):

δ (ppm) = 1.38, 1.74 (2 d, *J* = 6.8, 6.7 Hz, Σ = 3 H, **a**-, **b**-CH_3), 2.8-2.95 (br d, 1 H, OH, exchangeable with D_2O), 4.93-5.06, 5.08-5.20 (2 m, Σ = 1 H, **b**-, **a**- H), 5.43-5.58 (m, 1 H, 1-H), 6.88-7.1 (m, 2 H, aromat. H), 7.29-7.45 (m, 1 H, aromat. H).

MS (direct inlet, EI, 70 eV):

m/z (%) = 217 (11) [M^+], 199 (9) [M^+-H_2O], 171 (26) [M^+-NO_2], 170 (21) [M^+-HNO_2], 144 (27), 143 (69) [$F_2C_7H_4OH^+$, α-cleavage], 142 (90), 141 (100) [$F_2C_6H_3CO^+$], 114 (60), 113 (83) [$F_2C_6H_3^+$], 109 (38), 68 (80), 57 (59) [M^+-HNO_2-$F_2C_6H_3$, α-cleavage].

HRMS (direct inlet, EI, 70 eV): $C_9H_9F_2NO_3$: Calc. 217.0550, Found 217.0547.

3.14 Cathodic hydrodimerization of 16 to 30

Using the GWP (chapt. 3.1) (*E,E*)-2,5-dinitro-1,6-diphenylhexa-1,5-diene (**16**, 0.98 g, 3.02 mmol) are electrolyzed at −1.4 V until 863 C (2.96 F mol^{-1} corresponding to 1.48 F/mol

C=C-NO_2). 3,6-Dinitro-1,2-diphenylcyclohexane (**30**, 0.40 g, 1.23 mmol, 40%) was obtained as colourless, crystalline mixture of diastereomers. By flash chromatography the mixture can only partially separated; therefore the spectroscopic data are given for the mixture.

IR (KBr):

ν (cm^{-1}) = 3100, 3050 (=CH), 2990, 2950 (CH), 1545 (NO_2), 1455, 1380 (NO_2), 1340, 1030, 810, 755, 695 (=CH).

^{1}H NMR ($CDCl_3$):

δ (ppm) = 2.0-3.0 (m, 4 H, 2 CH_2), 3.3-4.2 (m, 2 H, benzylic H), 4.8-5.55 (m, 2 H, α-nitro-H), 6.7-7.9 (m, 10 H, aromat. H).

^{13}C NMR ($CDCl_3$, only one fraction sufficiently soluble):

δ (ppm) = 23.21, 26.18, 29.28, 29.29 (t, C-4/-5), 48.16, 50.46, 51.46, 51.57 (d, C- 1/-2), 83.44, 83.65, 84.47, 88.98 (d, C-3/-6), 127.70, 127.87, 128.26, 128.32, 128.48, 128.52, 128.60, 130.35 (d, aromat. C), 133.44, 135.56, 135.81, 136.15 (s, aromat. C-1).

MS (GC/MS-coupling, EI, 70 eV):

m/z (%.) = 326 (9) [M^+], 249 (14) [M^+-HNO_2-NO], 233 (23) [M^+-HNO_2 - NO_2], 232 (13) [M^+-2 HNO_2], 155 (15), 143 (16), 129 (36), 117 (58), 115 (48), 105 (28), 91 (100) [$C_7H_7^+$], 77 (8), 65 (9), 51 (6).

$C_{18}H_{18}N_2O_4$ (326.4) Calc. C 66.25, H 5.56, N 8.58; Found: C 66.36, H 5.72. N 8.48

3.15 Cathodic hydrodimerization of 17 to 31

Using the GWP (chapt. 3.2.1) 1-nitrocyclohex-1-ene (**17**, 0.65 g, 5.11 mmol) were electrolyzed at −1.3 V until 563 C (1.14 F mol^{-1}) were consumed. 1-(2-Nitrocyclohex-1-yl)-2-nitrocyclohexane (**31**, 0.45 g, 1.76 mmol, 68%) were obtained as colourless, crystalline mixture of diastereomers. Flash chromatography affords two fractions with different composition of diastereomers. ^{1}H NMR spectroscopy indicates in the different chemical shifts of the α-nitromethine protons four different diastereomers with a ratio of about **31a**:**b**:**c**:**d** = 38:9:14:1.

IR (KBr):

ν (cm^{-1}) = 2910, 2840 (CH), 1540, 1525 (NO_2), 1435, 1365 (NO_2), 1345, 1235, 1100, 985, 885, 850, 820, 720.

^{1}H NMR ($CDCl_3$):

31a: δ (ppm) = 0.85-2.2 (m, 16 H, 8 CH_2), 2.2-2.4 (m, 2 H, O_2NCHC*H*C*H*CHNO_2), 4.37 (dt, *J* = 4.0, 11.2 Hz, 1 H, O_2NC*H*CHCHCHNO_2), 4.62-4.69 (m, 1 H, O_2NCHCHCHC*H*NO_2).

31b: δ (ppm) = 0.85-2.2 (m, 16 H, 8 CH_2), 2.2-2.4 (m, 2 H, O_2NCHC*H*C*H*CHNO_2), 4.76 (dt, *J* = 4.11 Hz, 1 H, O_2NC*H*CHCHCHNO_2), 5.01-5.09 (m, 1 H, O_2NCHCHCHC*H*NO_2).
31c+**d**: δ (ppm) = 1.0-1.4 (m, 6 H, 3 CH_2), 1.7-2.0 (m, 10 H, 5 CH_2), 2.28- 2.35 (m, 2 H, O_2NCHC*H*C*H*CHNO_2), 4.26, 4.46 (2 dt, *J* = 4.11 Hz, 2 H, **d**-,**c** O_2NC*H*CHCHC*H*NO_2).
^{13}C NMR ($CDCl_3$):
31a: δ (ppm) = 19.78, 20.82, 24.21, 24.69, 25.16, 25.26, 30.24, 32.52 (8 t, diastereotopic ring-CH_2), 40.57, 43.26 (2 d, O_2NCH*C*HCHCHNO_2), 86.14, 89.53 (2 d, O_2N*C*HCHCH*C*HNO_2).
31b: δ (ppm) = 19.71, 23.75, 23.95, 2425, 24.89, 25.34, 30.53, 31.93 (8 t, diastereotopic ring-CH_2), 40.96, 44.60 (2 d, O_2NCH*C*H*C*HCHNO_2), 81.24, 88.00 (2 d, O_2N*C*HCHCH*C*HNO_2).
31c: δ (ppm) = 23.90, 24.19, 24.67, 31.95 (4 t, ring-CH_2), 41.18 (d, O_2NCH*C*H*C*HCHNO_2), 87.27 (d, O_2N*C*HCHCH*C*HNO_2).
31d: δ (ppm) = 23.89, 25.01, 27.62, 33.28 (4 t, ring-CH_2), 43.11 (d, O_2NCH*C*HCHCHNO_2), 87.69 (d, O_2N*C*HCHCH*C*HNO_2).
MS (GC/MS-coupling, EI, 70 eV):
m/z (%) = 256 (0) [M^+], 210 (0.2) [M^+-NO_2], 179 (10) [M^+-HNO_2-NO], 163 (48) [M^+-HNO_2-NO_2], 162 (21) [M^+-2 HNO_2], 128 (3) [M+/2], 121 (12), 95 (72), 81 (100) [$C_6H_9^+$, mid cleavage and HNO_2-split off], 79 (26) [$C_6H_7^+$], 69 (24), 67 (61), 55 (24), 41 (37).
$C_{12}H_{20}N_2O_4$ (256 3) Calc. C 56.24, H 7.86, N 10.93; Found: C 56.05, H 7.77, N 10.91.

4 References

[1] Hass, H. B.; Susie, A. G.; Heider, R. L. *J. Org. Chem.* **1950**, *15*, 8-14. doi:10.1021/jo01147a002
[2] Gairaud, C. B.; Lappin, G. R. *J. Org. Chem.* **1953**, *18*, 1-3. doi:10.1021/jo01129a001
[3] Autorenkollektiv, Organikum, 15. Ed., VEB Deutscher Verlag der Wissenschaften, Berlin 1984, p. 567.
[4] Boberg, F.; Garburg, K.-H.; Görlich, K.-J.; Pipereit, E.; Ruhr, M. *Liebigs Ann. Chem.* **1984**, 911-919. doi:10.1002/jlac.198419840510
[5] Padeken, H. G.; von Schickh, O.; Segnitz, A. *Methoden Org. Chem. (Houben-Weyl)* 4. Ed. 1971, *Vol. X/1*, p. 342.
[6] Padeken, H. G.; von Schickh, O.; Segnitz, A. Methoden Org. Chem. (Houben-Weyl) 4. Ed. 1971, G. Thieme, Stuttgart, Vol. X/1, p. 351.
[7] Padeken, H. G.; von Schickh, O.; Segnitz, A. Methoden Org. Chem. (Houben-Weyl) 4. Ed. 1971, G. Thieme, Stuttgart, Vol. X/1, p. 355.
[8] Stille, J. K.; Vessel, E. D. *J. Org. Chem.* **1960**, *25*, 478-480. doi: 10.1021/jo01073a623

[9] Lipina, E. S.; Perekalin, V.; Bobovich, Y. S. *J. Gen. Chem. USSR (Engl. Transl.)* **1964**, *34*, 3683-3687.
[10] Sonn, A.; Schellenberg, A. *Ber. Dtsch. Chem. Ges*. **1917**, *50*, 1513-1525. doi:10.1002/cber.19170500251
